# Deleterious mitochondrial DNA point mutations are overrepresented in *Drosophila* expressing a proofreading-defective DNA polymerase γ

**DOI:** 10.1371/journal.pgen.1007805

**Published:** 2018-11-19

**Authors:** Colby L. Samstag, Jake G. Hoekstra, Chiu-Hui Huang, Mark J. Chaisson, Richard J. Youle, Scott R. Kennedy, Leo J. Pallanck

**Affiliations:** 1 Molecular and Cellular Biology Program, University of Washington, Seattle, WA, United States of America; 2 Department of Genome Sciences, University of Washington, Seattle, WA, United States of America; 3 Department of Pathology, University of Washington, Seattle, WA, United States of America; 4 Surgical Neurology Branch, National Institute of Neurological Disorders and Stroke, National Institutes of Health, Bethesda, MD, United States of America; 5 Computational Biology and Bioinformatics, University of Southern California, Los Angeles, CA, United States of America; MRC Mitochondrial Biology Unit, University of Cambridge, UNITED KINGDOM

## Abstract

Mitochondrial DNA (mtDNA) mutations cause severe maternally inherited syndromes and the accumulation of somatic mtDNA mutations is implicated in aging and common diseases. However, the mechanisms that influence the frequency and pathogenicity of mtDNA mutations are poorly understood. To address this matter, we created a *Drosophila* mtDNA mutator strain expressing a proofreading-deficient form of the mitochondrial DNA polymerase. Mutator flies have a dramatically increased somatic mtDNA mutation frequency that correlates with the dosage of the proofreading-deficient polymerase. Mutator flies also exhibit mitochondrial dysfunction, shortened lifespan, a progressive locomotor deficit, and loss of dopaminergic neurons. Surprisingly, the frequency of nonsynonymous, pathogenic, and conserved-site mutations in mutator flies exceeded predictions of a neutral mutational model, indicating the existence of a positive selection mechanism that favors deleterious mtDNA variants. We propose from these findings that deleterious mtDNA mutations are overrepresented because they selectively evade quality control surveillance or because they are amplified through compensatory mitochondrial biogenesis.

## Author summary

The energy needs of an animal cell are supplied by tiny organelles known as mitochondria. Each of the many mitochondria in a cell has a set of blueprints for making more mitochondria, known as mitochondrial DNA (mtDNA). As animals age, their mtDNA acquires irreversible defects called mutations, which accumulate and may cause aging. Cells can selectively destroy malfunctioning mitochondria, so we hypothesized that mitochondria with harmful mutations would be selectively destroyed. To test our theory, we created a fruit fly strain with a high mtDNA mutation rate. Our hypothesis predicts that, because mitochondria bearing harmful mtDNA mutations would be destroyed, we should detect primarily less harmful mutations in our strain. However, the mtDNA mutations we detected were more harmful than expected by chance. We suggest two possible explanations: First, mitochondria with harmful mtDNA mutations may be degraded less often because they generate little energy and are not damaged by toxic byproducts of energy production. Second, cells may compensate for harmful mtDNA mutations by stimulating mitochondria to multiply, creating more healthy mitochondria but also more mitochondria with harmful mtDNA mutations. Future studies will distinguish between these models and further advance our understanding of aging and aging related disease.

## Introduction

Mitochondria contain the electron transport chain complexes responsible for generation of most of a cell’s energy and also play crucial roles in Ca^2+^ buffering, metabolite synthesis, and apoptosis [1–3]. In addition to the ~1,000–2,000 nuclear genes that encode mitochondrial proteins, mitochondria contain a separate small circular genome, densely packed with 37 genes, that is essential for mitochondrial function. Mitochondrial DNA (mtDNA) mutations transmitted through the female germline are responsible for a host of incurable mitochondrial syndromes [4]. In addition, accumulation of mtDNA mutations in somatic tissues is implicated in aging [5, 6] and common diseases of the elderly including cancer [7] and neurodegenerative diseases [8]. There are typically thousands of copies of the mitochondrial genome in a single cell, such that when mtDNA mutations occur, they frequently share residence with wild-type mtDNA, a condition known as heteroplasmy. High levels of heteroplasmic mutations correlate with the severity of mitochondrial diseases [9], yet we know little about the factors that influence the frequency of mtDNA mutations or the emergence of their associated phenotypes [10, 11].

To explore the cellular mechanisms that influence the frequency and pathogenicity of mtDNA mutations, we are using the fruit fly *Drosophila* as a model system. In previous work, we found that many fundamental features associated with somatic mtDNA mutations in mammals are conserved in *Drosophila*, including a similar mtDNA mutation frequency, a preponderance of transition mutations, and an increased frequency of mtDNA mutations with age [12]. Bratic et al. further extended the utility of using *Drosophila* to study mtDNA mutations by knocking in exonuclease- and polymerase-deficient forms of DNA polymerase γ (PolG), the polymerase responsible for replicating the mitochondrial genome [13]. While these mutant strains are developmentally lethal in the larval stage as homozygotes, heterozygotes for the exonuclease-deficient PolG exhibited increased mtDNA mutation frequency. However, the physiological effects of this high mutation burden and the possibility of negative selection acting against the resulting mtDNA mutations were not explored.

In our current work, we created a transgenic proofreading-deficient version of the *Drosophila* mtDNA polymerase (designated PolG^mut^) that confers a 10- to 55-fold increase in the mtDNA mutation frequency, depending on transgene dosage without exhibiting embryonic lethality. PolG^mut^ expressing flies exhibited dosage-dependent phenotypes analogous to those of human mitochondrial diseases, including shortened lifespan, a progressive locomotor defect, and loss of dopaminergic neurons. Analysis of the frequency and distribution of mtDNA mutations in these mutator flies revealed an unexpectedly high ratio of nonsynonymous to synonymous mtDNA mutations. The mutations detected in mutator flies also tended to occur preferentially at conserved mtDNA sequences and resulted in pathogenic alterations. Together, these findings suggest that positive selection acts in favor of deleterious mitochondrial variants, either through the selective evasion of mutant-bearing mitochondria from negative selection or because cells that stochastically acquire deleterious mtDNA mutations activate compensatory mitochondrial biogenesis. Future work with this mtDNA mutator model will facilitate the study of cellular mechanisms that influence the frequency and pathogenesis of mtDNA mutations, as well as the identification of molecular factors that influence these processes.

## Results

### Generation of a *Drosophila* mtDNA mutator strain

**Fig 1:**
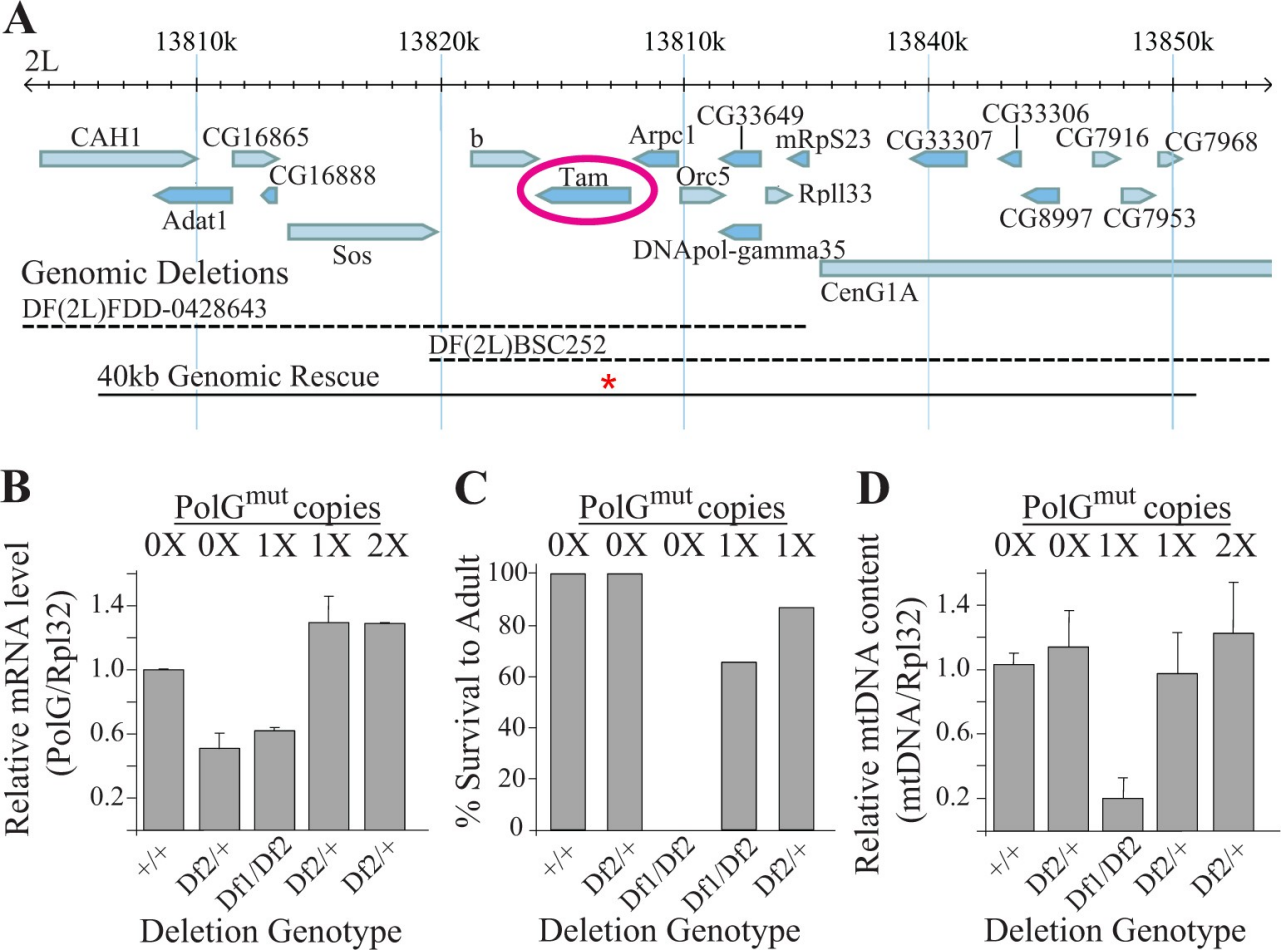
Transgenic expression of an exonuclease-deficient PolG rescues the larval lethality caused by overlapping deletions that remove *Drosophila PolG*. **(A)** The genomic region containing the *Drosophila PolG* gene *tamas* (*tam*). Blue bars represent known or predicted transcripts. Dashed lines correspond to sequences eliminated by the deletion alleles *Df(2L)FDD-0428643* (DF1) and *Df(2L)BSC252* (DF2). The solid black bar corresponds to the 40kb transgenic construct containing the D263->A263 mutation in *Drosophila* PolG (designated by the red asterisk), which spans the overlap between the *Df(2L)FDD-0428643* and *Df(2L)BSC252* deletions. **(B)** Total RNA was extracted and qPCR was used to measure PolG and Rpl32 transcript abundance from 7-day-old flies of the given genotype (n = 4 per genotype, three independent replicates) and their ratios are indicated. **(C)** The percentage of larvae of the given genotype that survived until the adult stage of development is indicated (n≥236 flies per genotype). **(D)** DNA was extracted from 7-day-old flies and qPCR was used to measure COX1 and Rpl32 DNA abundance from flies of the given genotype (n = 2 7-day-old male flies per genotype, three independent replicates) and their ratios are indicated. Error bars in panels B and D represent standard error.

Previous work has shown that altering a conserved aspartate residue in the second exonuclease domain of PolG to alanine impairs proofreading ability and results in a dramatically elevated mtDNA mutation frequency in multiple species [13–17]. Thus, we generated a *Drosophila PolG* transgenic construct with an alanine substitution at the equivalent site (designated *PolG*^*mut*^; Fig 1A). Because overexpression of *PolG* in *Drosophila* results in mtDNA depletion [18], we created our transgene using a genomic DNA fragment containing both the endogenous *PolG* gene and its associated *cis*-regulatory transcriptional elements to avoid artifacts associated with overexpression (Fig 1A). This construct was then used to create transgenic flies using standard methodologies (see Materials and Methods).

Flies bearing one or two copies of the *PolG*^*mut*^ transgene expressed similar levels of *PolG* mRNA as the endogenous *PolG* gene, thus confirming that this transgene does not cause PolG overexpression (Fig 1B). Moreover, a single copy of the *PolG*^*mut*^ transgene was capable of rescuing the recessive lethal phenotype caused by an overlapping set of deletions that remove the endogenous *PolG* gene, thus confirming that the *PolG*^*mut*^ transgene encodes a functional mtDNA polymerase (Fig 1A and 1C). However, the rescued flies displayed a marked reduction in mtDNA copy number (Fig 1D), consistent with previously published work suggesting that *Drosophila* PolG bearing this proofreading alteration is not fully functional [13]. In contrast, flies expressing one or two copies of the *PolG*^*mut*^ transgene in a strain hemizygous for the endogenous *PolG* gene did not display mtDNA depletion (Fig 1D). Given that mtDNA depletion could potentially confer phenotypes that are unrelated to mtDNA mutations, all of our remaining work involved the use of flies hemizygous for the endogenous *PolG* gene (Df2) and bearing zero, one, or two copies of the *PolG*^*mut*^ transgene, which we refer to as 0xPolG^mut^, 1xPolG^mut^, and 2xPolG^mut^, respectively.

**Fig 2:**
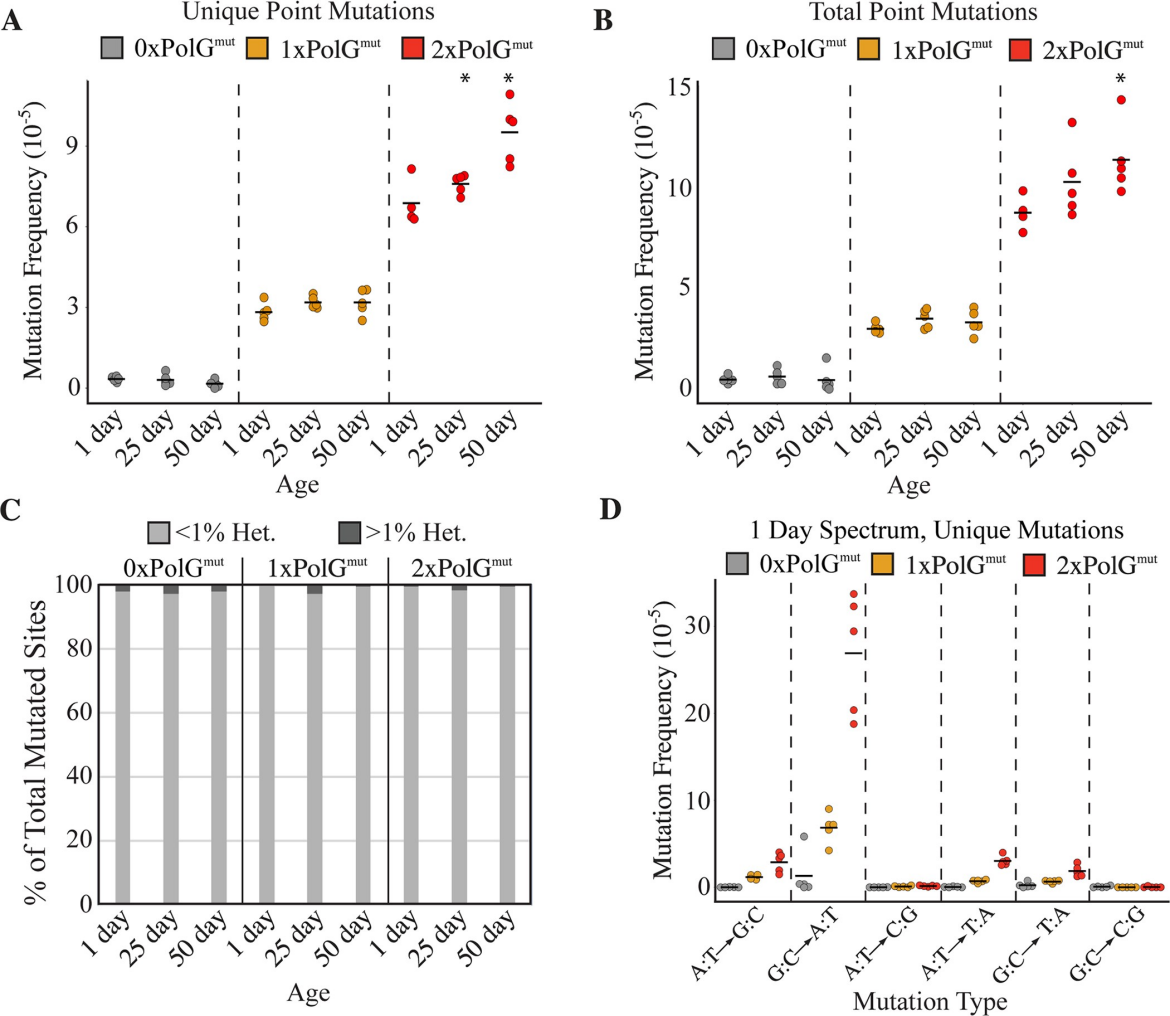
Transgenic expression of an exonuclease-deficient PolG results in a dose-dependent increase in mutation frequency. The frequencies of base substitution mutations were quantified in 1-day-old, 25-day-old, and 50-day-old flies of the given genotype using DS. **(A)** The mutation load of unique mutations, representing each unique mutation counted once, thus reflecting the *de novo* somatic mutation frequency. **(B)** The frequency of total mutations. **(C)** The percentage of mutated sites at sub-clonal levels (<1% heteroplasmy) and clonally expanded mutations (≥1% heteroplasmy) in flies of the indicated age and genotype. **(D)** The frequency of unique mutations of each type of base substitution mutation is indicated in 1-day-old flies of the indicated genotypes. N = 5 per genotype per time point. Horizontal bars in panels A, B, and D represent the mean frequency of the indicated group. *p < 0.05 compared to 1-day-old flies of the same genotype by Wilcoxon rank-sum test. 1x and 2xPolG^mut^ flies displayed significantly elevated mutation frequencies compared to age-matched control 0xPolG^mut^ flies at all time points (p<0.05 by Wilcoxon rank-sum test).

To test whether the *PolG*^*mut*^ transgene conferred an increased mtDNA mutation frequency, we performed Duplex Sequencing (DS) on mtDNA isolated from individual heads of 1-day-old transgenic flies. DS is a high-accuracy next-generation sequencing approach capable of detecting a single mutation in >10^7^ wild-type bases [19]. We addressed the possible influence of genetic background by comparing *PolG*^*mut*^ transgenic flies to control non-transgenic flies hemizygous for the endogenous *PolG*. Furthermore, fly strains were outcrossed to the same WT strain prior to sequence analysis to ensure that all of the genotypes being compared inherit their mtDNA from the same parental strain, as well as to replace mitochondrial genomes that had potentially accumulated mtDNA mutations over multiple generations of replication by the mutator polymerase (S1 Fig). We first tested whether the *Polg*^*mut*^ transgene introduces greater replication errors by measuring the frequency of unique mutations (see materials and methods for description of unique mutations) in 1-day-old flies. 0xPolG^mut^ (control) flies had a mutation frequency comparable to the frequency previously reported for WT flies (3.4x10^-6^ ± 8.9x10^-7^). By contrast 1xPolG^mut^ flies exhibited significant increases in the point mutation, insertion and deletion frequency relative to controls (Fig 2A, S4 Fig), and the frequency of these mutations increased further in 2xPolG^mut^ flies (Fig 2A, S3 Fig, S4 Fig). Because 2xPolg^mut^ flies inherit one copy of the *PolG*^*mut*^ transgene maternally (S1 Fig), the increased mtDNA mutation frequency in 2x relative to 1xPolG^mut^ animals may derive from an increased somatic mtDNA mutation frequency, as well as mutations that arise in the female germline. Consistent with previous work involving exonuclease-deficient mtDNA polymerases, mutator flies exhibited an increased prevalence of predominantly G:C to A:T transition mutations (Fig 2D) [14, 20, 21].

We next explored the influence of age on mtDNA mutation frequency in mutator flies by sequencing mtDNA from flies aged 25 and 50 days. The highest mtDNA mutation frequencies were observed in 50-day-old 2xPolG^mut^ flies, in which the point mutation frequency of unique mutations was elevated ~55-fold relative to age-matched controls (Fig 2A). Although there was a trend towards increased mutation frequency with age for all mutation types detected, only 2xPolG^mut^ flies exhibited a significant age-associated increase in point mutations relative to young flies of the same genotype (Fig 2A). Like young mutator flies, aged mutator flies also exhibited a prevalence of G:C to A:T transition mutations (S2 Fig). Additionally, when we combined data from control 0xPolG^mut^ animals of all ages to increase the total number of mutations detected, G:C to A:T transition mutations were also the most frequent mutation type detected, consistent with our previously published work [12].

**Fig 3:**
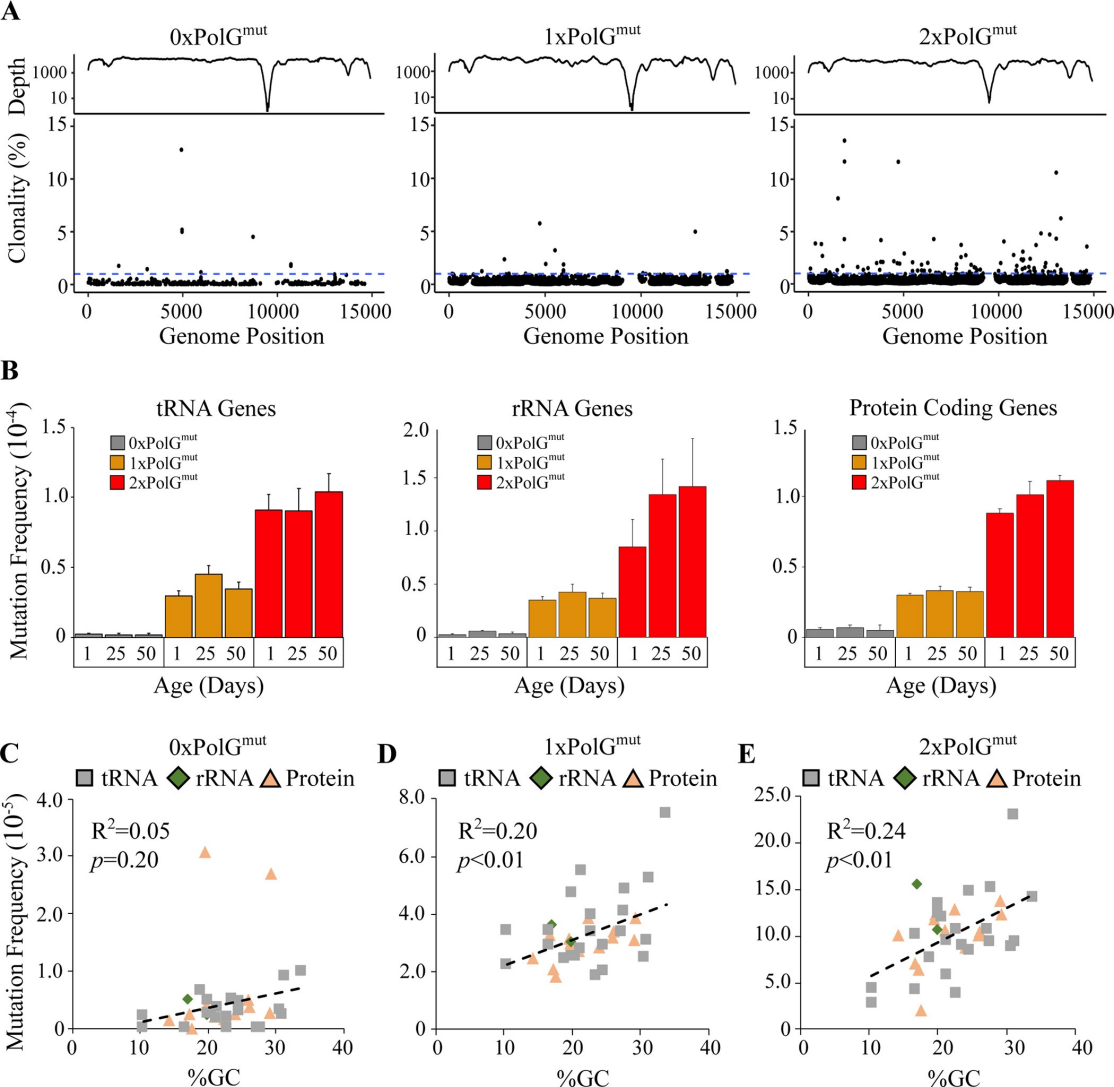
Mutation frequency is relatively uniform across the mitochondrial genome. **(A)** Sequencing depth (log_10_-transformed y-axis) and percent heteroplasmy of mutations across the mitochondrial genome in flies of all ages for the indicated genotype. 1% heteroplasmy is indicated by the dashed blue line. **(B)** Mutation frequency of mitochondrial tRNAs, rRNAs and protein coding genes for flies of the given genotype and age are shown. The correlation between GC content and mutation frequency in **(C)** tRNA, **(D)** rRNA, and **(E)** protein coding sequences for flies of all ages in the genotypes indicated. *p*-value was determined by Pearson correlation.

To search for evidence of clonal expansion, we also quantified the total mutation frequency, which in contrast to the unique mutation frequency, includes multiple occurrences of the same mutation. Control flies harbor a low total mutation burden (1-day-old flies had just 4.7x10^-6^ ± 1.8x10^-6^ mutations/nucleotide, or ~1 point mutation per 14 mtDNA molecules; Fig 2B, S1 Table) that is similar to that of the unique mutation frequency, suggesting little clonal expansion occurs in WT flies [12]. The total mutation frequency increased in a dose-dependent manner in mutator flies: 1-day-old 1xPolG^mut^ flies had a mutation frequency of 3.0x10^-5^ ± 0.4x10^-5^ (~one point mutation per 2.2 molecules of mtDNA Fig 2B, S1 Table) and 1-day-old 2xPolgmut flies had a mutation frequency of 8.8x10^-5^ ± 0.9x10^-5^ (~1.3 point mutations/mtDNA molecule; Fig 2B, S1 Table). Although the total mutation frequency in mutator flies exceeded that of the unique mutation frequency, the increase was small and we discovered very few clonally-expanded mutation sites in flies of any genotype (Fig 2C). Furthermore, very few mutations rose above 1% heteroplasmy even in old 2xPolG^mut^ flies (Figs 2C and 3A). These findings suggest that clonal expansion of mtDNA mutations is either restricted to individual cells, or that the short lifespan of *Drosophila* is incompatible with extensive clonal expansion of mtDNA mutations.

Our previous work to measure the mtDNA mutation frequency in *Drosophila* involved the Random Mutation Capture method, which only allowed us to analyze three small parts of the mitochondrial genome, thus precluding detailed analysis of the distribution of mtDNA mutations [12]. By contrast, DS enabled us to characterize the frequency of mutations across the entire mitochondrial coding sequence. Only the non-coding control region [ChrM:14917–19524] was refractory to DS owing to its high A:T content (~95%), which prevents efficient sequence capture and accurate reassembly. We found that mutations were distributed relatively uniformly between tRNA, rRNA, and protein-coding genes with no apparent mutational hotspots or mutational deserts (Fig 3A and 3B, S3 Fig). The mild variation in mutation frequency detected between genes is likely explained by differences in GC content and the G:C to A:T mutation bias of the polymerase (Fig 3C).

### Mutator flies exhibit reduced longevity, progressively worsening locomotor ability, neurodegeneration and mitochondrial dysfunction

**Fig 4:**
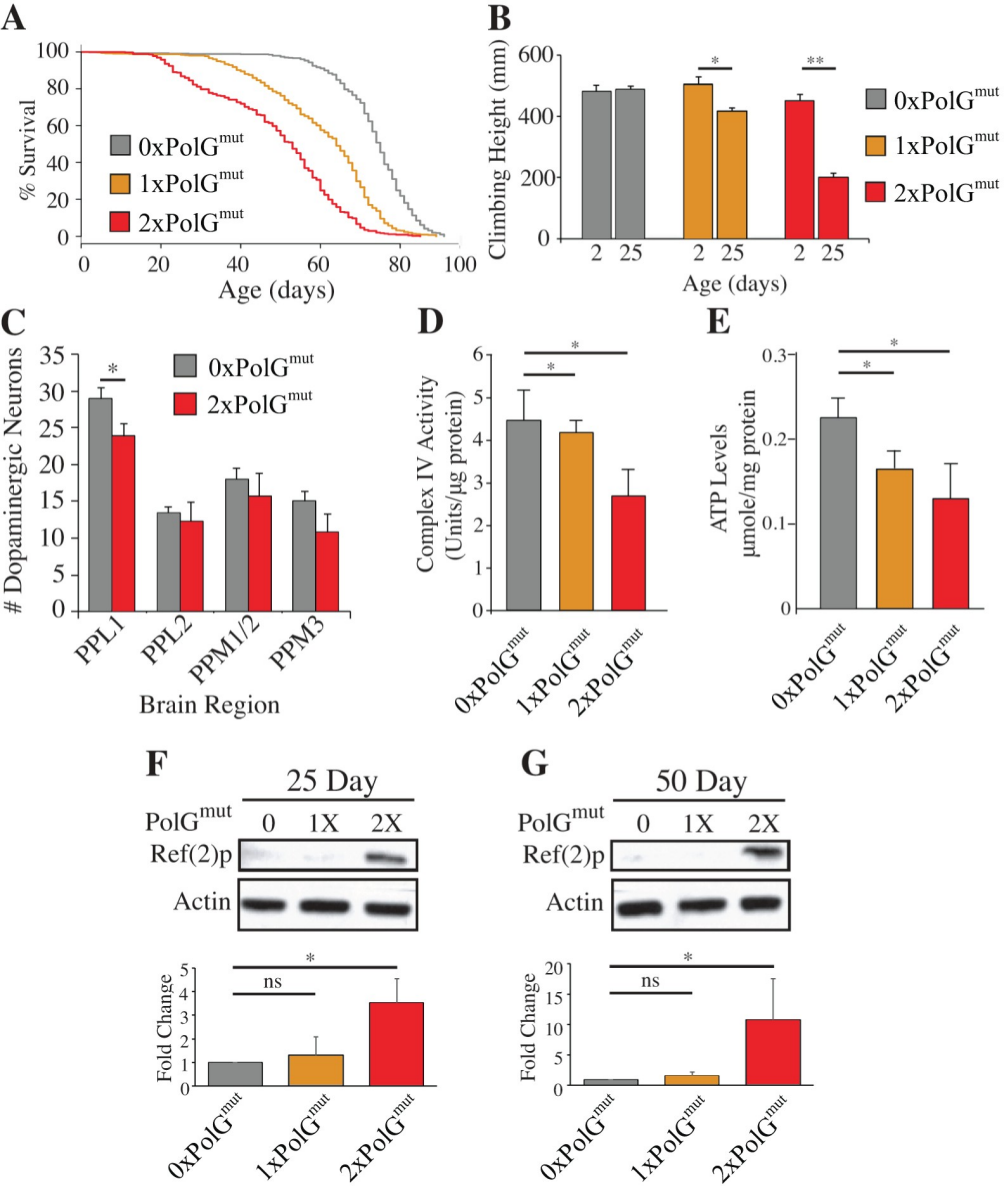
mtDNA mutator flies exhibit shortened lifespan, a locomotion defect, neuron loss, and mitochondrial dysfunction. **(A)** The lifespan of male flies of the indicated genotypes are shown (n≥364 flies per genotype). *p < .001 determined by log-rank test. **(B)** The mean distance climbed by male flies of the given ages and genotypes in 3 seconds (n≥54 flies per genotype per time point). **(C)** Quantification of dopaminergic neurons within the indicated clusters of in 50-day-old control and 2xPolG^mut^ flies. PPL1, protocerebral posterior lateral; PPM, protocerebral posterior medial **(D)** Complex IV activity is inversely correlated with PolG^mut^ dosage in 14-day-old flies (n = 4 male flies per genotype). **(E)** ATP abundance is similarly decreased in 14-day-old 1xPolg^mut^ and 2xPolg^mut^ flies. **(F) and (G)** Ref(2)P/p62 abundance is selectively increased in 25- and 50-day-old 2xPolg^mut^ flies, respectively. Error bars in panels B and C represent standard deviation, error bars in panels D-G represent standard error. *p < 0.05; **p < 0.01 by Student’s t-test.

Mutator mice display premature aging phenotypes, including a reduced lifespan, kyphosis, and hair loss [15]. Therefore, we tested whether mutator flies also exhibit signs of premature aging by examining lifespan and locomotor activity. Control flies had a median lifespan of 75 days, whereas the 1xPolG^mut^ flies had a modest reduction in lifespan, displaying a median lifespan of 64 days (Fig 4A). 2xPolG^mut^ flies showed a further reduction in lifespan, with a median lifespan of 53 days. Mutator flies also displayed a defect in locomotor performance using a simple test of climbing ability. Normal flies exhibit negative geotaxis, climbing to the top of a vial after being tapped to the bottom, and this behavior declines naturally as flies age. The presence of the *PolG*^*mut*^ transgene did not influence climbing ability in young flies, but conferred a dose-dependent decline in climbing behavior in older flies relative to controls (Fig 4B). The decline in climbing ability preceded the onset of decreased viability in 2xPolG^mut^ flies and ultimately culminated in a complete failure in climbing at ages >25 days. Approximately 10% of 2xPolG^mut^ flies also exhibited a rhythmic seizure phenotype beginning approximately 24 hours prior to death (S1 Video). This phenotype was not observed in any other genotype.

A number of previous observations indicate that dopaminergic neurons are particularly sensitive to mitochondrial stress [22–25]. In particular, mitochondrial toxins and mutations affecting mitochondrial quality control pathway components result in the selective death of dopaminergic neurons in humans and animal models [22]. Moreover, a recent study has shown that mice expressing a proofreading-deficient form of DNA polymerase γ exhibit selective dopaminergic neuron death when crossed to *parkin* mutant mice [26]. These observations prompted us to test whether an increased load of mtDNA mutations leads to degeneration of dopaminergic neurons in *Drosophila*. To perform this analysis, we dissected whole brains and immunostained with antiserum to tyrosine hydroxylase to quantify the number of dopaminergic (DA) neurons in 50-day-old flies. The number of DA neurons in the protocerebral posterior lateral 1 (PPL1) cluster was significantly reduced in 2xPolG^mut^ flies (Fig 4C, S5 Fig), suggesting that a high mtDNA mutational load triggers the loss of a subset of dopaminergic neurons consistent with these prior reports [27].

To test whether the premature aging phenotypes of mutator flies are caused by mitochondrial dysfunction, we monitored several mitochondrial functional parameters. Since PolG mutator mice show reduced Complex IV activity and assembly [26], we assayed Complex IV activity in mutator flies and found it to be significantly reduced in a dose-dependent fashion relative to age-matched controls (Fig 4D). Consistent with this deficit, mutator flies also had a reduced abundance of ATP (Fig 4E). 2xPolG^mut^ flies also had elevated levels of the autophagy marker Ref(2)p (the *Drosophila* homolog of p62), possibly suggesting that there is a buildup of autophagic intermediates in response to an upregulation of autophagy (Fig 4F and 4G). However, the abundance of the mitochondrial unfolded protein stress markers HSP60 and mitochondrial HSP70 were unchanged in mutator flies (S6 Fig), indicating that the increased load of mtDNA mutations does not result in sufficient protein misfolding to trigger activation of the mitochondrial unfolded protein stress response. We also observed an abnormal downturned wing posture in 2xPolG^mut^ mutator flies at 35 days of age, similar to that seen in *Drosophila PINK1* and *parkin* mutants [28]. However, in contrast to *PINK1* and *parkin* mutants, both of which exhibit apoptotic muscle degeneration, there was no gross evidence of muscle degeneration or apoptosis in mutator flies. Ultrastructural examination of flight muscle tissue also failed to detect alterations in mitochondrial morphology or integrity (S7 Fig). Together, these results suggest that flies harboring high mutation loads suffer from non-structural muscle abnormalities, thus potentially making these flies a suitable model for the study of mitochondrial myopathies.

### Deleterious mtDNA mutations are overrepresented in mutator flies

Studies in cultured cells have indicated the existence of pathways that can be manipulated to decrease the frequency of a deleterious heteroplasmic mutation [29–32]. However, there is little evidence that these pathways are normally operative in the somatic tissues of an intact animal model. To address this matter, we subjected mutator flies to a variety of analyses aimed at the detection of selective forces acting against harmful mutations. To account for the clonality of mutations, we used the frequency of total mutations in all of our remaining analyses. To diminish the influence of sampling bias and increase the number of mutations detected per animal, we re-sequenced 1-day-old 1xPolG^mut^ flies using a technical advance in reagent preparation for the Duplex Sequencing protocol that was developed during the course of our study, thus providing us with a high-quality dataset with very high sequencing depth. The use of 1xPolG^mut^ flies for this analysis also ensures that the mutations detected from sequencing are acquired in somatic tissues (S1 Fig).

**Fig 5:**
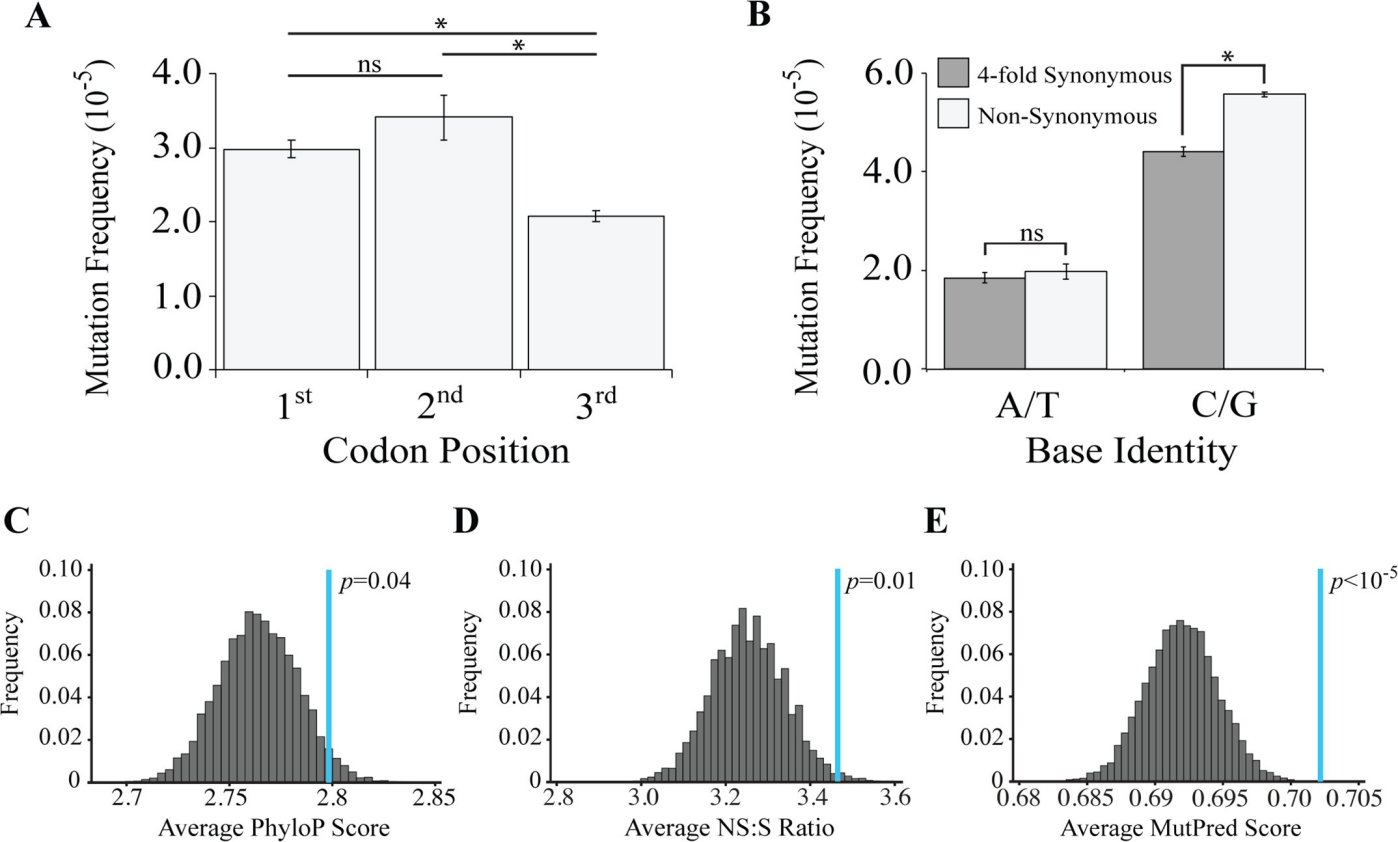
Mutator flies accumulate an excess of deleterious mtDNA mutations. **(A)** The mutation frequency of 1-day-old 1xPolG^mut^ flies per codon position. **(B)** The mutation frequency at four-fold degenerate (synonymous; S) and non-synonymous (NS) sites. **(C)** The distribution of PhyloP scores from 10,000 trials of simulated mutagenesis of the *Drosophila* mitochondrial genome under conditions of neutrality. The blue line indicates the observed average PhyloP score from 1-day-old 1xPolG^mut^ flies. **(D)** The distribution of NS/S ratios observed from 10,000 trials of simulated mutagenesis of the *Drosophila* mitochondrial genome under conditions of neutrality. The blue line indicates the observed average NS/S ratio in 1-day-old 1xPolG^mut^ flies. **(E)** The distribution of MutPred scores from 10,000 trials of simulated mutagenesis of the *Drosophila* mitochondrial genome under conditions of neutrality. The blue line indicates the observed average MutPred score in 1-day-old 1xPolG^mut^ flies. *p*-values for A and B determined through pooled sample Z-test. *p*-values in panels C-E determined empirically.

Because the third codon position is often degenerate, we hypothesized that negative selection acting against deleterious variants would result in a lower frequency of mutations at the first two codon positions relative to the third codon position. Our results were in complete reverse to our expectations: we detected higher mutation frequencies at the first and second codon positions (Fig 5A). However, a confound in this analysis concerns the high frequency of mutations at G:C base pairs, and the relative deficiency of G:C base pairs in the third codon position (S8 Fig). Notably, the AT-rich *Drosophila* mitochondrial genome, like many other insect species, primarily consists of A:T bases at four-fold degenerate (i.e., synonymous) sites, where the frequency of A:T base pairs is 94% [33, 34]. By contrast, G:C base pairs are predominantly located at nonsynonymous (NS) sites. To circumvent this confound, we compared the mutation frequency at NS sites and at four-fold synonymous (S) sites separately for A:T and G:C base pairs. Because mutations arising at four-fold degenerate sites do not alter the encoded amino acid, such mutations should be present at higher frequency relative to those at NS sites in the context of negative selection acting against deleterious variants. We detected no significant difference in the mutation frequency between NS sites and S sites at A:T positions. However, at G:C positions the mutation frequency was higher at NS sites relative to S sites, in complete opposition to the expectations of a negative selection model (Fig 5B).

The unexpected finding that deleterious mtDNA variants were overrepresented in *Drosophila* prompted us to examine this matter further. Specifically, we performed Monte Carlo simulations of random mutagenesis, such that we could compare our findings from sequencing mutator flies to a neutral mutational model derived from simulations. Because the mutation frequency at G:C sites greatly exceeds that at A:T sites, we performed simulations to precisely mirror the mutational biases detected in mutator flies. Each round of simulation selected the proportion of each type of mutation detected within protein-coding regions of 1xPolG^mut^ flies and computationally redistributed these mutations randomly throughout the coding portions of the mtDNA. Moreover, because the probability of detecting a mutation at any given site in the mitochondrial genome is directly proportional to the sequencing depth at that site, we also weighted the probability of detecting a nucleotide alteration at each site according to sequencing depth at that site in our simulations (for further details, see the Materials and Methods section). Simulations were repeated 10,000 times to create a distribution of neutral outcomes, thus providing a framework to compare findings from 1xPolG^mut^ flies.

We performed three analyses designed to detect whether negative selection reduces the frequency of pathogenic mtDNA mutations. First, we tested the hypothesis that mutations at evolutionarily conserved mtDNA sites are more susceptible to negative selection than those at sites of low conservation. Second, we tested the hypothesis that mutations resulting in nonsynonymous (NS) alterations are more prone to negative selection than synonymous (S) mutations. Third, we tested the hypothesis that mutations resulting in deleterious amino acid alterations are more susceptible to negative selection than those that result in conservative amino acid alterations. Our hypotheses predict that mtDNA mutations arising at highly conserved sites, particularly those mutations that result in deleterious NS amino acid alterations, are eliminated through negative selection. Consequently, these deleterious mutations should be underrepresented in mutator flies relative to a distribution of randomly generated mutations.

To test the prediction that mutations at conserved sites are underrepresented in mutator flies, we used the PhyloP algorithm, which calculates mtDNA positional conservation scores from pairwise comparisons between 27 insect species [35]. The PhyloP algorithm assigns a logarithmic score to each nucleotide position indicating the degree of evolutionary conservation at that site; a score of zero indicates neutral evolution, whereas negative and positive scores suggest accelerated evolution and increased conservation, respectively. We then compared the average PhyloP scores from the mutations identified in mutator flies to a distribution of PhyloP scores created from Monte Carlo simulations of random mutagenesis. Consistent with our comparison of NS and four-fold S sites, we found that mutations at sites with high PhyloP scores are overrepresented, again suggesting that deleterious variants are overrepresented in mutator flies (Fig 5C).

We next tested whether NS mutations were underrepresented in mutator flies by calculating the NS/S ratio and comparing this ratio to a distribution of NS/S ratios obtained from Monte Carlo simulation of random mutagenesis (neutrality). Our hypothesis that negative selection preferentially eliminates nonsynonymous mutations predicts a reduction in the occurrence of nonsynonymous mutations and thus the observed NS/S ratio should be lower than expected from neutrality. In contrast to our hypothesis, the NS/S ratio detected in mutator flies is significantly elevated relative to neutrality, indicating that nonsynonymous mutations are overrepresented in mutator flies (Fig 5D). These findings are inconsistent with the hypothesis that negative selection acts against nonsynonymous mutations.

As a final test of the model that negative selection acts against deleterious mtDNA variants, we used the MutPred algorithm [36] to compare the pathogenicity of nonsynonymous mutations found in mutator flies to a distribution of MutPred scores created from Monte Carlo simulations of random mutagenesis. The MutPred algorithm uses the structural and functional properties of a protein to predict the functional consequence of a nonsynonymous amino acid substitution, and previous work has established the validity of the MutPred algorithm to predict the consequences of mtDNA mutations [37–39]. MutPred assigns scores ranging from 0 to 1 to quantify the pathogenicity of a particular variant, with higher scores indicating a greater likelihood of pathogenicity. If negative selection acts to preferentially remove the most deleterious mutations, mutator flies should accumulate variants with low MutPred scores. In contrast to this prediction, the detected mutations have an average MutPred score that is significantly higher than expected from a neutral mutational model (Fig 5E). To confirm findings from simulations, we added the combined data from our previously acquired low-depth sequencing of 1x and 2xPolG^mut^ flies in an effort to increase the total number of mutations. We then reran our simulations using this data and applied the same three metrics to ask if there is selection against harmful mutations. Results of this analysis again revealed that deleterious mutations are overrepresented in mutator flies (S10 Fig).

Our previous work revealed a strand asymmetry in the occurrence of C:G to T:A mutations [12], and this phenomenon could potentially influence our distributions of simulated mutations. To eliminate the potential confound of a mutational strand asymmetry, we repeated our analysis using the coding sequence of the *Mitochondrial Cytochrome c oxidase subunit I* (*COX1*) gene, which does not exhibit strand asymmetry in the mutation spectrum (S9A Fig). While we did not detect a significant difference in the evolutionary conservation of mutations arising in *COX1* (S9B Fig) relative to neutrality, we discovered that these mutations displayed an elevated NS/S ratio (S9C Fig) and an overrepresentation of mutations with high MutPred Scores (S9D Fig), indicating that our findings are not a consequence of mutational strand asymmetry. Taken together, our analyses do not support the model that selection acts against deleterious mtDNA mutations; instead, our findings indicate that pathogenic mtDNA variants are overrepresented in mutator flies.

### The trinucleotide context of mutation sites does not explain the overrepresentation of deleterious mutations

**Fig 6:**
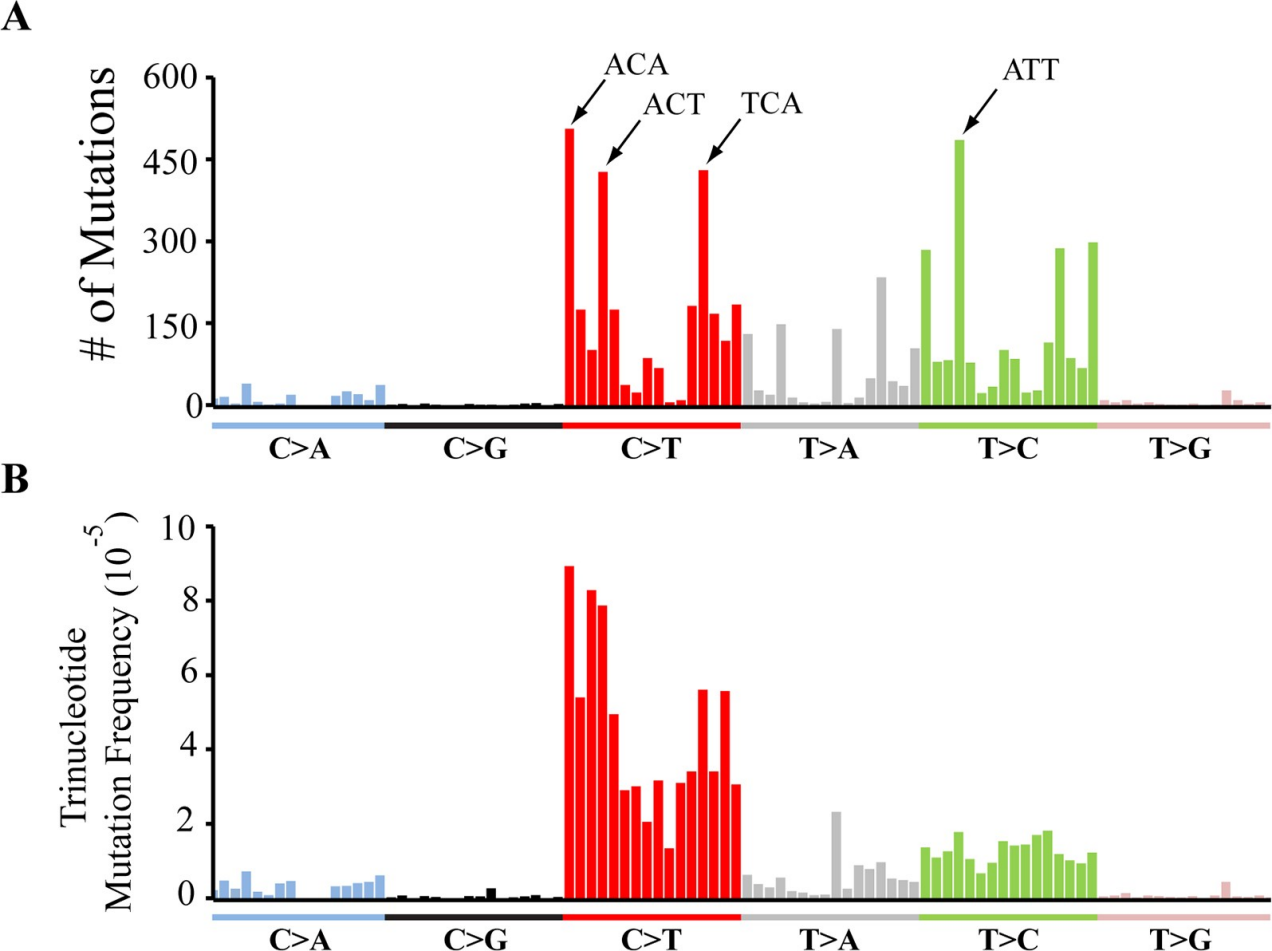
The trinucleotide context of mutations does not explain the overrepresentation of deleterious mutations. **(A)** The raw number of mutations detected in 1xPolG^mut^ flies within all 96 possible trinucleotide contexts. **(B)** The trinucleotide mutation frequency at all 96 possible trinucleotide contexts. Trinucleotide contexts are presented in the order previously characterized [77], and the four most highly mutated contexts annotated in panel A.

One potential explanation for the overabundance of deleterious mutations in mutator flies is that nucleotide context influences the mutation frequency and, by chance, results in a higher mutation frequency at functionally important sites. Such an occurrence was observed in mutator mice where the high abundance of C>T transitions within the ‘TCA’ context was suggested to explain the high pathogenicity of mtDNA mutations in this organism [40]. To explore this model, we examined the influence of trinucleotide context of the nucleotides directly 5’ and 3’ to the mutation bearing sites on mutation frequency. We first began by computing the number of mutations observed in all 96 trinucleotide contexts. Like mutator mice, we found that C>T mutations within the TCA context represented one of the most abundant trinucleotide mutation categories. However, unlike mutator mice, the TCA context was not the most abundant mutation category and the abundance of mutations within the TCA context was similar to the frequency of other C>T mutation contexts (Fig 6A). Also, despite the high frequency of G:C>A:T mutations, trinucleotide contexts associated with T>C transitions, such as the ATT trinucleotide context, were similarly abundant to those associated with G:C>A:T mutations (Fig 6A).

A limitation of comparing the total abundance of mutations within a particular trinucleotide context is that this value will be influenced by the prevalence of that context in the genome. In addition, our ability to detect mutations at any given site in the genome will depend on sequencing depth at that site. Thus, to test whether the trinucleotide context influences mutation frequency, we calculated the trinucleotide mutation frequency by normalizing the raw number of mutations observed in each trinucleotide context to their prevalence in the genome and to the sequencing depth at these sites (see Materials and Methods). Analysis of the trinucleotide mutation frequency substantially decreased the heterogeneity across the entire C>T trinucleotide context (Fig 6B). Normalizing our data in this fashion also revealed that the high abundance of particular T>C mutation contexts reflects the composition of the *Drosophila* mitochondrial genome rather than an influence of sequence context on mutation frequency (Fig 6B). Given the similarity of the mutation frequency within any given trinucleotide context, we conclude that the trinucleotide context does not play a major role in the overrepresentation of deleterious mutations.

## Discussion

The progressive accumulation of deleterious mtDNA mutations in somatic tissues is implicated in aging and common diseases of the elderly. Although the frequency of these mutations correlates with the severity of the symptoms that they cause [9], little is known about the pathways that influence the frequency and pathogenicity of mtDNA mutations. In previous work, we showed that many of the features associated with mtDNA mutations are similar in flies and vertebrates, including a low frequency of mutations and a preponderance of transition mutations [12, 13]. Our current work offers further evidence in support of these previous findings by using a high accuracy next-generation sequencing approach that allowed us to sensitively detect mtDNA mutations over a broader region of the mitochondrial genome [19]. We have also extended the utility of *Drosophila* as a model system for studying mtDNA mutations by creating a *Drosophila* mtDNA mutator strain that exhibits a dramatically increased mtDNA mutation frequency and displays features associated with mitochondrial diseases and premature aging including mitochondrial dysfunction, reduced lifespan, a progressive locomotor deficit, and loss of dopaminergic neurons. Surprisingly, deleterious mtDNA mutations were overrepresented in mutator flies, suggesting the existence of a novel selective mechanism underlying this phenomenon. Our current work provides a foundation to explore the factors responsible for the overabundance of harmful mtDNA mutations and the pathways that influence the pathogenicity of these mutations.

Our findings warrant comparison to another recently described *Drosophila* mtDNA mutator strain that was created by introducing the same exonuclease mutation used in our current study into the endogenous *PolG* locus [13]. While heterozygotes for this *PolG* knock-in allele displayed a similar increase in the mtDNA mutation frequency to flies that bear a single copy of our mutator transgene, homozygotes for the knock-in *PolG* allele did not survive beyond the larval stage of development. By contrast, a single copy of our *PolG*^*mut*^ transgene rescued the recessive lethal phenotype of an overlapping set of deletions that completely remove the endogenous *PolG* gene. Given that our mutator transgene appears to express endogenous levels of *PolG* transcript, we are at a loss to explain this discrepancy. Moreover, heterozygotes for the *PolG* knock-in allele displayed no obvious effect on lifespan [41], whereas our mutator lines displayed a dose dependent decrease in lifespan. The explanation for this difference is also unknown but may reflect differences in the mtDNA genetic background in these two studies, which we have found in unpublished work can influence lifespan. Further study of the knock-in *PolG* mutant revealed that the mtDNA mutation frequency progressively increased in subsequent generations, indicating that purifying selection is unable to fully keep pace with an increased mtDNA mutation rate [13]. Although we have not directly explored the influence of our mutator transgene in the female germline, we find that mutator stocks lose viability within several generations without periodic outcrossing, consistent with the model that the accumulation of mtDNA mutations is responsible for this loss of viability.

The finding that somatic mtDNA mutations accumulate with age at an accelerated rate relative to mutations in the nuclear genome has led to the suggestion that aging may be a consequence of accumulated mtDNA mutations [9]. While the phenotypes of homozygous PolG mutator mice were initially offered as support for this hypothesis, it was later found that heterozygous mutator mice live a normal lifespan despite having a mtDNA point mutation frequency that greatly exceeds that of elderly WT mice [42, 43]. Our findings also indicate that a high mtDNA point mutation load can negatively affect longevity. However, our data do not support the model that the shortened lifespan of mutator flies results from the progressive accumulation of mtDNA mutations. Although there is a trend towards increased mtDNA point mutation frequency with age in mutator flies, this age-dependent increase in mtDNA point mutations is small in comparison to the mutation frequency in young mutator flies. For example, the mutation frequency in 50-day-old 1xPolG^mut^ is approximately 19-fold higher than age-matched controls, but fully 89% of the mutations detected in 50-day-old 1xPolG^mut^ are acquired by the time these flies reach 1 day of age. These findings suggest that a high mtDNA mutational load throughout life simply increases the probability of death later in life. The delayed phenotypic effects of mtDNA mutations may reflect the long half-lives of mitochondrial proteins [29, 44–46], such that the consequences of a mtDNA mutation would require substantial time to develop. Additionally, our finding that most of the somatic mtDNA mutations in mutator flies are acquired during development, coupled with the fact that at least most of these mutations presumably result from replication errors, indicates that there is likely relatively little mtDNA replication in adult flies. This conclusion is further bolstered by the finding that key components of the mtDNA replication apparatus, including PolG, the Twinkle helicase, and the mitochondrial biogenesis factor Spargel, are primarily expressed in the female ovary, and early in development when most tissue growth occurs [47, 48]. The importance of early arising mtDNA mutations to aging phenotypes may be conserved in vertebrates. Young mutator mice also display a dramatic increase in mtDNA mutation frequency relative to controls, but the mtDNA mutation frequency increases little with age, consistent with the model that these mutations occur primarily during development [15].

Previous work testing whether negative selection acts against deleterious mtDNA mutations has led to conflicting findings. Studies in cultured cells have demonstrated that pharmacological and genetic perturbations that activate mitophagic pathways can select against certain severe heteroplasmic mtDNA mutations [29–31]. Work in the nematode *C*. *elegans* has also shown that autophagy is required for the elimination of radiation-induced mtDNA damage [49], and that inactivation of the mitophagy-promoting factor Parkin results in increased abundance of point mutations in a mitochondrial mutator background and increased abundance of a deleterious mtDNA deletion when the mitochondrial unfolded protein stress pathway is activated [50–52]. Overexpression of autophagy-promoting factors in *Drosophila* also reduced the frequency of a heteroplasmic deletion created by expression of a mitochondrially-targeted restriction endonuclease [53]. Studies in mice expressing a proofreading-defective mtDNA polymerase also indicate that mitochondrial turnover is increased relative to controls [54]. However, reducing the activity of PINK1 and Parkin in an otherwise WT *C*. *elegans* genetic background did not significantly influence the frequency of point mutations or a large mtDNA deletion, suggesting that this pathway does not ordinarily select against deleterious mtDNA mutations [50–52]. Moreover, mutator mice do not exhibit an altered NS/S mutation ratio relative to WT mice [20, 26, 55], and mutator mice lacking the mitophagy-promoting factor Parkin do not exhibit an increased mtDNA mutation frequency or an altered NS/S mutation ratio relative to mutator mice [26]. Previous work in *Drosophila* also suggests that negative selection does not act against a heteroplasmic mtDNA mutation in somatic tissues in the absence of extreme measures to induce autophagy [53, 56], and our current findings are fully consistent with this observation.

*In vitro* studies indicate that PINK1 and Parkin selectively target depolarized mitochondria for lysosomal degradation [57]. Thus, one possible explanation for the apparent absence of negative selection opposing the accumulation of deleterious mtDNA mutations is that the mitochondria that bear these mutations are not sufficiently depolarized to trigger activation of the PINK1-Parkin pathway *in vivo*. Even in the event of a mtDNA mutation sufficient to trigger severe depolarization, ATP synthase (Complex V) is capable of coupling the hydrolysis of ATP to maintain partial membrane potential [31, 58]. Alternatively, the fusion of mitochondria bearing mutations with healthy mitochondria containing WT genomes may allow deleterious mtDNA mutations to evade negative selection through genetic complementation. Indeed, mitochondrial stress frequently elicits mitochondrial fusion as a compensatory response [59]. While such compensatory mechanisms may prove useful when mtDNA mutations are present at low abundance, the phenotypes associated with high mutational loads in worms, flies, mice and humans indicate that these potential compensation pathways are incapable of fully preventing the deleterious consequences of a high mtDNA mutational load.

While we detect no evidence of negative selection acting against deleterious mtDNA mutations, a simple absence of negative selection does not fully explain our findings. If no selective forces were acting on mtDNA, the frequency of deleterious mtDNA mutations in mutator flies should match predictions from our simulations of neutrality. However, we found that deleterious mtDNA mutations were consistently overrepresented in mutator flies. One possible explanation for this finding is that there is positive selection for deleterious mtDNA mutations, which has previously been reported in vertebrates [60, 61]. Because many of the mtDNA mutations subjected to positive selection in vertebrates reside in or near the sequences that control mtDNA replication, it has been proposed that these mutations confer a replicative advantage, thus accounting for their overrepresentation [61]. However, a recent report has found strong evidence of positive selection acting on mutations that reside in protein coding sequences of human mtDNA with many of these variants appearing to result in deleterious effects on protein function [60]. One potential explanation for the excess accumulation of deleterious mutations in mtDNA coding sequences is offered by the “survival of the slowest model” [62]. In brief, this model posits that the mitochondrial quality control apparatus selectively targets oxidatively damaged mitochondria for degradation. According to this model, mitochondria that bear defective genomes are less metabolically active than fully functional WT mitochondria, and therefore less prone to damage from reactive oxygen species. This in turn makes these mutant-bearing mitochondria less prone to targeted degradation by quality control surveillance relative to fully functional mitochondria.

There are also at least two alternative models to explain the overabundance of deleterious mtDNA mutations in mutator flies. One possible explanation is that the exonuclease-deficient polymerase induces mutations in a sequence context-dependent fashion and that these contexts are enriched at critical residues. Although our analyses do not offer support for this model, we only examined the nucleotides immediately neighboring mutation sites, leaving open the possibility that other features of sequence context explain the overabundance of deleterious mutations in mutator flies. A second alternative model to explain the overabundance of deleterious mtDNA mutations in mutator flies is that the subset of cells that acquire deleterious mtDNA mutations may compensate for the presence of these defective genomes by inducing mitochondrial biogenesis. If the mitochondrial biogenesis machinery is incapable of distinguishing between mitochondria with defective and WT genomes, this phenomenon would inadvertently result in a higher replication frequency of deleterious mutations relative to benign mutations because replication would be selectively induced in cells that bear deleterious mutations. Although we do not detect overt evidence of increased mitochondrial biogenesis in mutator flies (Fig 1D), our model posits that mitochondrial biogenesis would only be induced in a subset of cells, and this modest level of induction may not be readily detectable on a macroscopic level. Two recent studies in *C*. *elegans* offer potential support for this model by showing that worm strains bearing a heteroplasmic mtDNA deletion maintain tight regulation of WT mtDNA abundance, but that the abundance of the deletion can vary dramatically between individuals in the population [50, 51]. These findings suggest that mutant genomes can “hitchhike” to high frequency as a consequence of a compensatory mitochondrial biogenesis response to decreased mitochondrial activity. However, a difference between these studies and our current work is that the mitochondrial unfolded protein stress pathway appears be an active participant, if not the driver, of the high mtDNA deletion frequency in *C*. *elegans* [50, 51], whereas we detected no evidence of mitochondrial unfolded protein stress pathway activation in mutator flies (S6 Fig). Future experiments will be required to more fully investigate the potential role of the mitochondrial unfolded protein stress pathway and other candidate pathways that may influence the frequency of deleterious mtDNA mutations in mutator flies.

## Materials and methods

### Fly strains and animal husbandry

All experiments were performed with flies raised and maintained at 25°C on standard cornmeal-molasses food unless otherwise stated. The *w*^*1118*^ isogenic fly strain, *Df(2L)BSC252* strain, and *Df(2L)FDD-0428643* strain were obtained from the Bloomington Drosophila Stock Center.

### Generation of the PolG^mut^ transgenic fly

The *Drosophila* POLG genomic region was PCR amplified from genomic DNA with primers (5’- TAAATCAATGTGACCGCCGC and 5’- TGTCCTTGCCTTGGGAACTG) and cloned into TOPO vector (Invitrogen). The D263A mutation was introduced by PCR using primers (5’- CAATGTCTCCTACGCAAGGGCGCGACTGAAG and 5’- CTTCAGTCGCGCCCTTGCGTAGGAGACATTG). Kanamycin resistance selection cassette (loxp-kanamycin-loxp) was PCR amplified and cloned into C-terminus of dPOLG-D263A using the PmeI site on the TOPO vector.

PCR primers containing 70 bp homology arms were used to generate linearized fragment for recombineering (5’-TACGGAGGAGTGTGTGGTCGCAGGTTGGACTTCAGTTGCCTTAAAGGATGTTTCCTTTATTAAAACGAGGATGCAGTTCCACCTGATCAG and 5’- TGCCTTGGGAACTGGGAAACGTATCGGCAACAGGATGCTTTAAATGCAAGGTTATTTAAAAACATAGTGATGTACAAGAAAGCTGGGTCG). The recombineering and Kanamycin excision process were performed using a published procedure [63, 64].

To increase transgenesis efficiency, a 40 kb P[acman] clone with dPOLGD263A was generated from modified 100 kb P[acman] constructs. In brief, two 500 bp homology arms flanking the POLG 40 kb fragment were PCR amplified using primers (left arm: 5’- AGGCGCGCCTGTATTGCCTCAGCCGGTTG and 5’-CGCGGATCCTCGCTGTGTCGATAAGGAAC; right arm: 5’-CGCGGATCCGTTCGATTTGGTCAACCTGC and 5’- AACTTAATTAAGAGTCCAATGGGATTCCACA) and cloned into attB-P[acman]-ApR vector [63]. The 40 kb fragment with dPOLGD263A was then introduced into attB-P[acman]-ApR by recombineering. A list of the genes situated on this 40 kb fragment, along with their presumed functions is shown in S3 Table.

To increase the copy number and allow for transgenesis, DNA from confirmed colonies was extracted and transformed into EPI300 cells. To create the PolG^mut^ transgenic fly, modified 40 kb P[acman] constructs were integrated specifically into the 92F1 and 28E7 regions of the *Drosophila* genome using a φ31-mediated transformation protocol (BestGene Inc., Rainbow Transgenic Flies, Inc.).

### Lifespan analysis

One- to two-day-old flies were collected into vials in groups of up to 20 flies. Flies were transferred every two to three days onto fresh food and the number of deaths was recorded. Lifespans were repeated at least three times with a minimum of 350 flies per genotype. The seizure phenotype of moribund mutator flies was noted during the lifespan analysis. All survivorship data were calculated using R software. The R package “survival” was used to generate Kaplan-Meier survival curves. Genotypes were compared using the log-rank test to determine significance.

### Analysis of climbing

Climbing was assayed using a modified protocol for the previously published rapid iterative negative geotaxis (RING) assay [65, 66]. Briefly, clean vials containing up to 20 files were placed into the RING apparatus. Flies were manually tapped to the bottom of the vials and their climbing behavior recorded using a digital video camera. This procedure was repeated for a total of three trials per vial and a minimum of 60 flies per genotype at each time point. Still images were captured using iMovie software (iMovie '11 v9.0.8. Apple Inc.) 3.0s after the flies were tapped down. The vertical height attained by each fly was scored using Fiji software (an open-source image processing software) [67]. Genotypes were compared to age-matched controls using the Wilcoxon rank-sum test.

### ATP measurements

Four adult flies were homogenized in 200 μl of lysis buffer (100 mM Tris, 4 mM EDTA) followed by flash freezing in liquid nitrogen. Samples were boiled for 3 min before centrifugation at 8000xg for 5 min. Supernatant was then diluted 50-fold with lysis buffer for ATP quantification. ATP levels were measured using a commercial ATP Determination Kit (Invitrogen) as previously described [68].

### Complex IV assay

Cytochrome *c* oxidase was measured as previously described [69]. Homogenates were prepared from 4 male flies homogenized with a hand-held rotor (VWR) in PBS with 0.1% Triton X-100 and protease inhibitor cocktail (Roche). Absorbance was measured with a SpectraMax M2 plate reader (Molecular Devices). Activities were normalized to total protein, and quantified using DC protein assay (Bio-Rad).

### Western blot analysis

To generate protein extracts for Western blot analyses, four male flies were collected and frozen in liquid nitrogen. Flies were thawed and manually homogenized using a pestle in 100μL of lysis buffer (50 mM Tris-HCl (pH 7.4), 150 mM NaCl, 1% NP-40, 10% glycerol, 10 mM NaF, 1 mM Na3VO4, 100 μg/ml PMSF, Sigma protease inhibitor cocktail (Sigma #P8340)). Cell debris was removed from the lysate through centrifugation at 13,000rpm for 15 minutes at 4°C. The protein lysate was boiled in SDS-PAGE sample buffer with 2% beta-mercaptoethanol for 10 minutes, and the resulting proteins subject to Western blot analysis.

For Western blot analyses, extracts were separated by SDS-PAGE on 4–20% Bis-Tris gels (GenScript #M42012) and transferred onto PVDF membranes overnight. Membranes were blocked in iBind Flex Solution Kit (SLF2020) and Western blots were performed using the iBind Flex Western Device (SLF2000) according to the manufacturer’s protocol. Immunodetections were performed using the following antibodies: 1:8000 mouse anti-Actin (#MAB1501, Chemicon/Bioscience Research Reagents), 1:200 rabbit anti-Ref(2)P (Ab178440, Abcam), 1:500 rabbit anti-HSP60 (#4870S, Cell Signaling Technology), 1:1000 rabbit anti-GRP 75/mt-Hsp70 (sc-13967, Santa Cruz Biotechnology, Inc.). The secondary antibody anti-mouse HRP (BioRad) was used at 1:1000 for actin. The secondary antibody anti-rabbit HRP (BioRad) was used at 1:2000 for Ref(2)P, and at 1:500 for HSP60 and GRP 75/mt-Hsp70. Signal was detected using Thermo Scientific electrochemoluminescence reagents. Quantification of western blot images was performed using Fiji software [67]. Western blot data were normalized using log-transformation to stabilize variance before means were compared using Student t-test. Each experiment was repeated with at least three biological replicates.

### Transmission electron microscopy

TEM was performed as previously described with minor modifications [70]. Briefly, indirect flight muscles were dissected from 50-day-old control and 2xPolG^mut^ flies and placed in fixative containing 2.5% glutaraldehyde, and 2% paraformaldehyde in 0.1 M sodium cacodylate buffer, pH 7.4, and incubated overnight at 4 °C. Fixed tissues were then postfixed in 1% OsO4, dehydrated in an ethanol series, and embedded using Epon. Samples were subjected to ultra-thin sectioning at 70 nm and stained with 6% uranyl acetate and a Reynolds lead citrate solution before TEM examination. Grids were viewed using a JEOL JEM 1400 transmission electron microscope.

### Quantification of dopaminergic (DA) neurons

Adult brain dissection, fixation, immunohistochemistry, and imaging were performed as described previously [71]. DA neurons were labeled with anti-TH antiserum (1:50, Immunostar). Serial optical sections were taken at 1-μm intervals and the confocal image stacks were analyzed using Imaris software (Bitplane Inc). The number of TH-positive neurons within each of the major DA neuron clusters was determined by visual inspection of individual confocal Z-series images.

### DNA isolation for sequencing

One- to two-day-old male flies were collected and transferred into fresh vials every 2–3 days. Once flies reached the appropriate age for sequencing, heads were harvested using a razor blade, flash frozen in liquid nitrogen, and stored at -80°C. Total DNA was isolated from individual fly heads using the QIAamp DNA Micro isolation kit following the manufacturer’s instructions. DNA yield for a single head typically ranged between 20-30ng of total DNA.

### Duplex sequencing

Total DNA was prepared for DS using a previously described protocol [72] with several modifications. Briefly, ~20 ng of total DNA was sonicated in 60 μL of nuclease-free ddH_2_O using a Covaris AFA system with a duty cycle of 10%, intensity of 5, cycles/burst 100, time 20 seconds x 5, temperature of 4°C. After sonication, each sample was subjected to end-repair and 3’-dA-tailing using the NEBNext Ultra End-repair/dA-tailing kit (New England Biolabs) according to the vendor’s instructions. Each sample was then ligated with 2 μL of 15 μM DS adapters, prepared as described [72] using the NEBNext Ultra Ligation kit (New England Biolabs) according to the manufacturer’s instructions. Each sample was then cleaned of excess adapters using AgenCourt AmpureXP magnetic beads and PCR amplified, as previously described [72]. After library construction, mtDNA was enriched for sequencing by targeted DNA capture using IDT xGen Lockdown probes (Integrated DNA Technologies) specific for non-repetitive and non-low complexity regions of the *Drosophila* mitochondrial genome, as designated by RepeatMasker (http://www.repeatmasker.org), using the manufacturer’s instructions. Probe sequences are found in S2 Table. Duplex Sequencing adapters used in collecting data for analyzing mutation selection were chemically synthesized as a collaborative effort with Integrated DNA Technologies to develop a prototype synthesis method.

The captured DNA samples were sequenced on an Illumina NextSeq500 using 150bp paired-end sequencing. The resulting reads were aligned against the *Drosophila* genome (BDGP Release 6 + ISO1 MT/dm6) using the Burrows-Wheeler Aligner and Samtools [73] coupled with a custom software workflow described previously [72]. Reads not uniquely mapping to the mitochondrial genome were excluded from further analysis. Reads mapping to a large repetitive region [ChrM:5961..5983] were excluded from our analyses to avoid artifacts caused by misalignment. The breakpoints of the repetitive region were determined using the RepeatMasker Web Server v.4.0.6 [74]. Sequence data has been uploaded to the Sequence Read Archive (SRA) repository, and can be accessed at SRA accession PRJNA495611. A heteroplasmy cutoff of 70% was applied to filter polymorphisms from the reference genome. After processing, we called unique somatic mutations by counting every mutation only once at each position of the genome. Total mutation frequency counts all mutations detected, including multiple occurrences at the same site.

### Analysis of mtDNA mutation spectrum and trinucleotide context

Spectrum data, mutation frequency by codon position, mutation context by GC content, and four-fold degenerate site analyses were performed using scripts developed in Python v.2.7. Parsing of the mitochondrial genome as well as GC content analyses were performed using Biopython [75]. All measures of mutation frequency are calculated as a fraction:

mutation frequency = [total # mutations / total sequenced bases]

of the indicated mutation type. For calculations of trinucleotide mutation frequency, the denominator of this equation is calculated for each of the 96 trinucleotide contexts by tabulating the sequencing depth at each nucleotide in the protein-coding sequence and then grouping according to its 3’ and 5’ flanking nucleotides. Scripts to analyze trinucleotide sequence context as well as trinucleotide mutation frequency were developed in Python v.3.4, and modified from a previously published duplex sequencing workflow [76]. Circular plots of the distribution of mutations were generated using the R package, ‘circlize.’ Statistics were performed and graphs were generated in RStudio v1.1.383, Microsoft Excel for Mac v.16.10, and StataCorp Stata v.12.1.

### Analysis of mtDNA mutation selection

To search for evidence of selection, a bioinformatics workflow was developed in Python v.2.7 (https://github.com/csamstag/mito-mutations) to analyze mtDNA mutation data obtained from duplex sequencing, and to run simulations of mutagenesis mimicking the mutation spectra obtained from sequencing mutator flies. Data for simulations were obtained by aggregating the results from sequencing four 1-day-old 1xPolg^mut^ flies. Point mutations were identified as described above, except that multiple mutations of a given type at the same site were also included in our analyses. Monte Carlo simulations were performed to generate a distribution of random mutations for statistical comparison to experimental findings. To control for the observed mutational biases in our data, we designed our scripts to match parameters observed from sequencing mutator flies (i.e., the same number of G:C to A:T mutations, G:C to T:A mutations, *etc*.). To account for variation in sequencing depth, random mutations were generated using a probability function that was weighted according to the total read depth at each position. Each round of simulation mirrored the spectrum and number of mutations observed in the protein-coding regions of mutator flies. Simulations were repeated 10,000 times, and each simulation was analyzed using three metrics designed to detect selective forces.

To examine the relationship between mutation frequency and sequence conservation, we obtained the PhyloP scores for each position in the *Drosophila melanogaster* mitochondrial genome from the UCSC Genome Browser. These values were used to calculate the average PhyloP score of the mutations detected from sequencing mutator flies. Similarly, we calculated the average PhyloP values of the mutations identified from simulations performed as described above. Empirical *p*-values represent the fraction of time a simulation displayed an average PhyloP score greater than or equal to the average PhyloP score observed in mutator flies.

To test whether selection influences the frequency of NS variants, we compared the average NS/S ratio obtained from sequencing mutator flies to the NS/S ratios obtained from simulations. We performed simulations as described above and binned those mutations that map to coding sequences according to whether they induce NS or S alterations. We then calculated the average NS/S ratio for each simulation. The empirical *p*-value reflects the fraction of simulations in which the NS/S ratio was greater than or equal to the average NS/S ratio observed in 1xPolg^mut^ flies. A similar approach was used to compare the NS/S ratio in the *COX1* gene from sequencing mutator flies to the distribution of NS/S ratios from simulations of mutagenesis of the *COX1* gene. Simulated mutagenesis of the *COX1* gene was performed as described above, including adjustments for sequencing bias, and sequencing depth.

To test whether selection influences the frequency of pathogenic mutations, we used MutPred [36] software to calculate pathogenicity scores for all NS variants detected in mutator flies, as well as all NS mutations generated from simulations. The average MutPred score from mutator flies was then compared to a distribution of MutPred scores from simulations. The empirical *p*-value was determined as the fraction of simulations in which the average MutPred score was greater than or equal to the average MutPred score observed in mutator flies. A similar approach was used to analyze the pathogenicity of NS mutations occurring within the *COX1* gene. Adjustments were made to simulations to account for mutational bias and sequencing depth within the *COX1* gene.

## Supporting information

S1 Fig The crossing schemes used in our work. The crossing schemes used to generate control 0xPolG^mut^ (Cross #1), 1xPolG^mut^ (Cross #2), and 2xPolG^mut^ (Cross #3) flies. Flies were outcrossed to females from the same isogenic w1118 strain prior to sequencing to eliminate accumulated mutations and control for genetic background effects.
(PDF)

S2 Fig The spectra of point mutations in 1-, 25-, and 50-day-old flies. The frequency of each type of base substitution mutation for Unique and Total mutations observed in in **(A)** 1-day-old, **(B)** 25-day-old, and **(C)** 50-day-old flies of the indicated genotype, N = 5 flies per genotype per time point.
(TIF)

S3 Fig The distribution of mutations in mutator flies. Plots of mtDNA mutations identified in 0xPolG^mut^, 1xPolG^mut^ and 2xPolG^mut^ flies. The outermost track of each plot designates functional elements within the mitochondrial genome. Red = Protein coding; Blue = tRNA; Green = rRNA; Black = Control Region. The middle track of each plot depicts the sites where mutations were observed within animals of the age indicated. The innermost track depicts the log-transformed average sequencing depth for flies of the indicated genotype. Note the absence of sequence coverage in the AT-rich control region, as well as the region between ~(ChrM ~9100–9850) not efficiently captured in our sequencing.
(PDF)

S4 Fig Mutator flies show a dose-dependent increase in small insertion and deletion frequency. **(A)** The frequencies of insertion mutations were quantified in flies of the given ages and genotype using DS. **(B)** The frequencies of deletion mutations (≤5bp) were quantified in flies of the indicated ages and genotypes using DS. *p-*values determined by Student’s t-test.
(PDF)

S5 Fig Dopaminergic neuron loss in mutator flies. Representative confocal images of immunostained brains of 50-day-old **(A)** 0xPolG^mut^ and **(B)** 2xPolG^mut^ flies. Dopaminergic neurons were immunostained using tyrosine hydroxylase antiserum. The PPL1-2 and PPM1-3 clusters of dopaminergic neurons are indicated.
(PDF)

S6 Fig The abundance of the mitochondrial unfolded protein stress markers Hsp60 and mitochondrial Hsp70 are unchanged in 1xPolG^mut^ or 2xPolG^mut^ flies. Western blots of 25- and 50-day-old 1xPolG^mut^ (1X) and 2xPolG^mut^ (2X) flies. Actin was used as a loading control. Images are representative of four biological replicates.
(PDF)

S7 Fig Mutator flies do not have mitochondrial morphological alterations. Electron microscopy of indirect flight muscle of 50-day-old **(A)** 0xPolg^mut^ and **(B)** 2xPolg^mut^ flies does not reveal additional mitochondrial ultrastructural defects in mutator flies.
(PDF)

S8 Fig Third codon sites in protein-coding genes have reduced GC content. All protein-coding genes show depleted GC content in third codon positions, as calculated from the *Drosophila* reference genome.
(PDF)

S9 Fig Deleterious nonsynonymous mutations are overrepresented within the coding sequence of the *COX1* gene. **(A)** Mutations within the *COX1* coding sequence display no strand bias for the predominant mutation type, G:C to A:T transitions. **(B)** The distribution of average PhyloP scores of mutations in the *COX1* coding sequence expected under neutrality, calculated from 10,000 trials of simulated mutagenesis under conditions of neutrality. Only mutations observed within the *COX1* gene [ChrM 1474–3009] were used for Monte Carlo resampling in these simulations. The blue line indicates the average PhyloP score of mutations observed in the *COX1* coding sequence of 1-day-old 1xPolG^mut^ flies. **(C)** The distribution of average NS/S ratios of mutations in the *COX1* coding sequence expected under neutrality, calculated from 10,000 simulations of random mutagenesis as described above. The blue line indicates the average NS/S ratio of mutations observed in the *COX1* coding sequence of 1-day-old 1xPolG^mut^ flies. **(D)** The distribution predicted average MutPred scores of NS variants observed in the *Drosophila COX1* coding region under conditions of neutrality, calculated from 10,000 simulations of random mutagenesis as described above. The blue line indicates the observed average MutPred score in NS variants found in the *COXI* sequence in 1-day-old 1xPolG^mut^ flies. *p*-values in panels B-D determined empirically.
(PDF)

S10 Fig Deleterious nonsynonymous mutations are overrepresented in a pooled sample of all mutator flies. **(A)** The distribution of average PhyloP scores of mutations in the protein-coding sequence expected under neutrality, calculated from 10,000 trials of simulated mutagenesis under conditions of neutrality. Sequence data from 1x and 2xPolG^mut^ flies of all ages were aggregated and Monte Carlo resampling simulations were performed as above. The blue line indicates the average PhyloP score of all mutations observed in the 1xPolG^mut^ and 2xPolG^mut^ flies. **(B)** The distribution of average NS/S ratios of mutations expected under neutrality, calculated from 10,000 simulations of random mutagenesis from the combined sequence data of 1xPolG^mut^ and 2xPolG^mut^ flies. The blue line indicates the average NS/S ratio of mutations observed across 1xPolG^mut^ and 2xPolG^mut^ flies. **(C)** The distribution predicted average MutPred scores of NS variants calculated from 10,000 simulations of random mutagenesis as described above. The blue line indicates the observed average MutPred score in NS variants found in 1xPolG^mut^ and 2xPolG^mut^ flies. *p*-values were determined empirically.
(PDF)

S1 Video The seizure phenotype of mutator flies. A steady rhythmic seizure phenotype developed in approximately 10% of 2xPolG^mut^ flies in the 24 hours preceding death. This phenotype was never observed in control or 1xPolG^mut^ animals.
(M4V)

S1 Table Summary statistics from duplex sequencing of control, 1xPolG^mut^, and 2xPolG^mut^ flies. The total number of nucleotides sequenced, mutations identified, and genome-wide mutation frequency are shown for each fly for **(A)** uniquely mutated sites, and **(B)** total mutations. These sequencing data represent nucleotides for which sequence reads could be mapped to the *Drosophila* mtDNA reference sequence and duplex consensus strands could be constructed.
(PDF)

S2 Table Probe sequences used for sequencing of the mitochondrial genome. (XLSX)

S3 Table Genes contained within the PolG^mut^ transgene along with their presumed functions. (PDF)
