# Supplementary material for: Deleterious mitochondrial DNA point mutations are overrepresented in Drosophila expressing a proofreading-defective DNA polymerase γ
Source: PLoS Genet. 2018 Nov 19;14(11):e1007805. doi: 10.1371/journal.pgen.1007805 (PMC6289449; doi:10.1371/journal.pgen.1007805)
Supplement: S1 Fig — The crossing schemes used to generate control 0xPolGmut (Cross #1), 1xPolGmut (Cross #2), and 2xPolGmut (Cross #3) flies. Flies were outcrossed to females from the same isogenic w1118 strain prior to sequencing to eliminate accumulated mutations and control for genetic background effects. (PDF) [file pgen.1007805.s001.pdf]

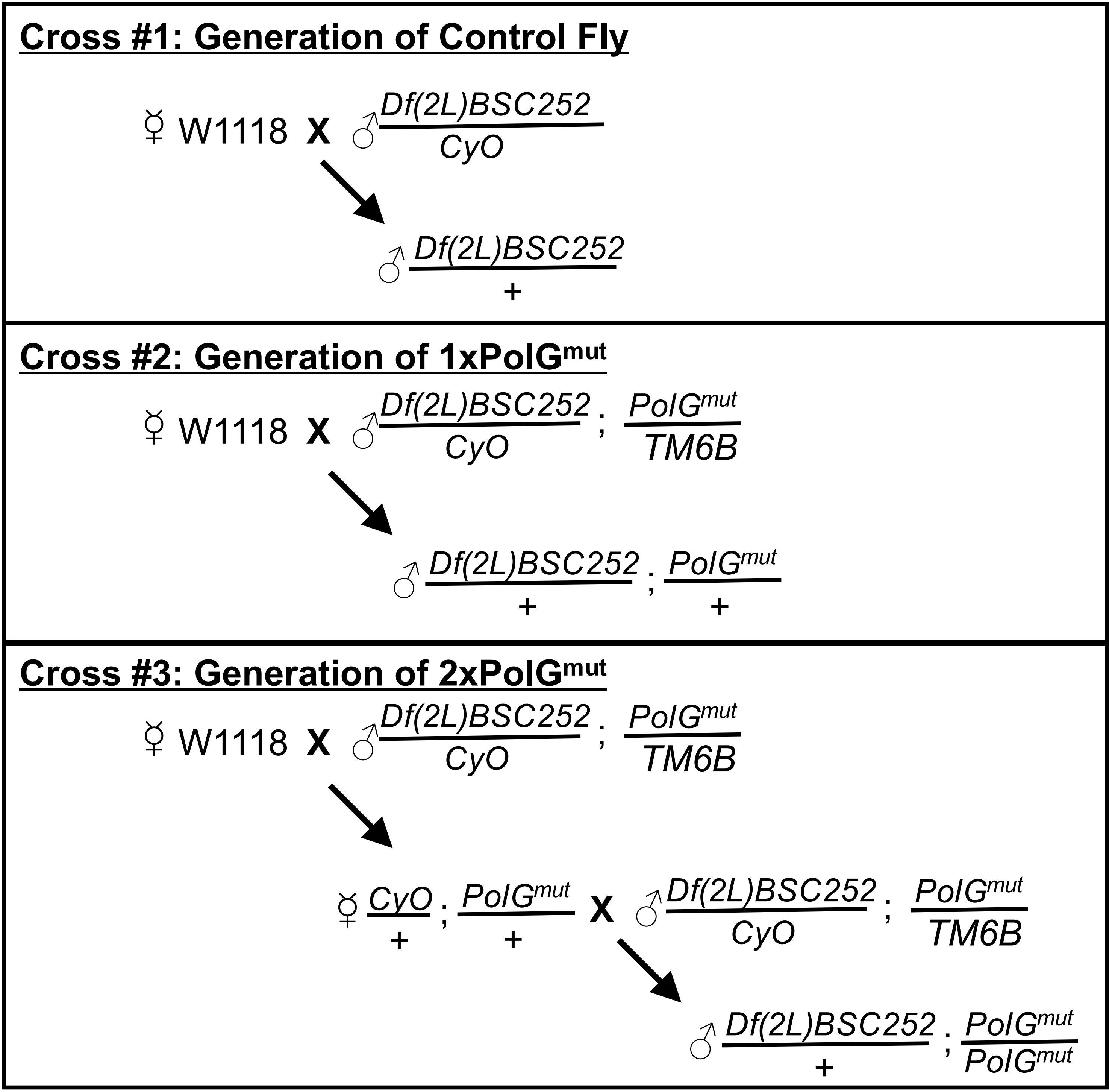

Cross #1: Generation of Control Fly
☿ W1118 X ♂ Df(2L)BSC252 / CyO
♂ Df(2L)BSC252 / +
Cross #2: Generation of 1xPolGmut
☿ W1118 X ♂ Df(2L)BSC252 / CyO ; PolGmut / TM6B
♂ Df(2L)BSC252 / + ; PolGmut / +
Cross #3: Generation of 2xPolGmut
☿ W1118 X ♂ Df(2L)BSC252 / CyO ; PolGmut / TM6B
☿ CyO / + ; PolGmut / + X ♂ Df(2L)BSC252 / CyO ; PolGmut / TM6B
♂ Df(2L)BSC252 / + ; PolGmut / PolGmut
