# Supplementary figures and images for: Deleterious mitochondrial DNA point mutations are overrepresented in Drosophila expressing a proofreading-defective DNA polymerase γ
Source: PLoS Genet. 2018 Nov 19;14(11):e1007805. doi: 10.1371/journal.pgen.1007805 (PMC6289449; doi:10.1371/journal.pgen.1007805)

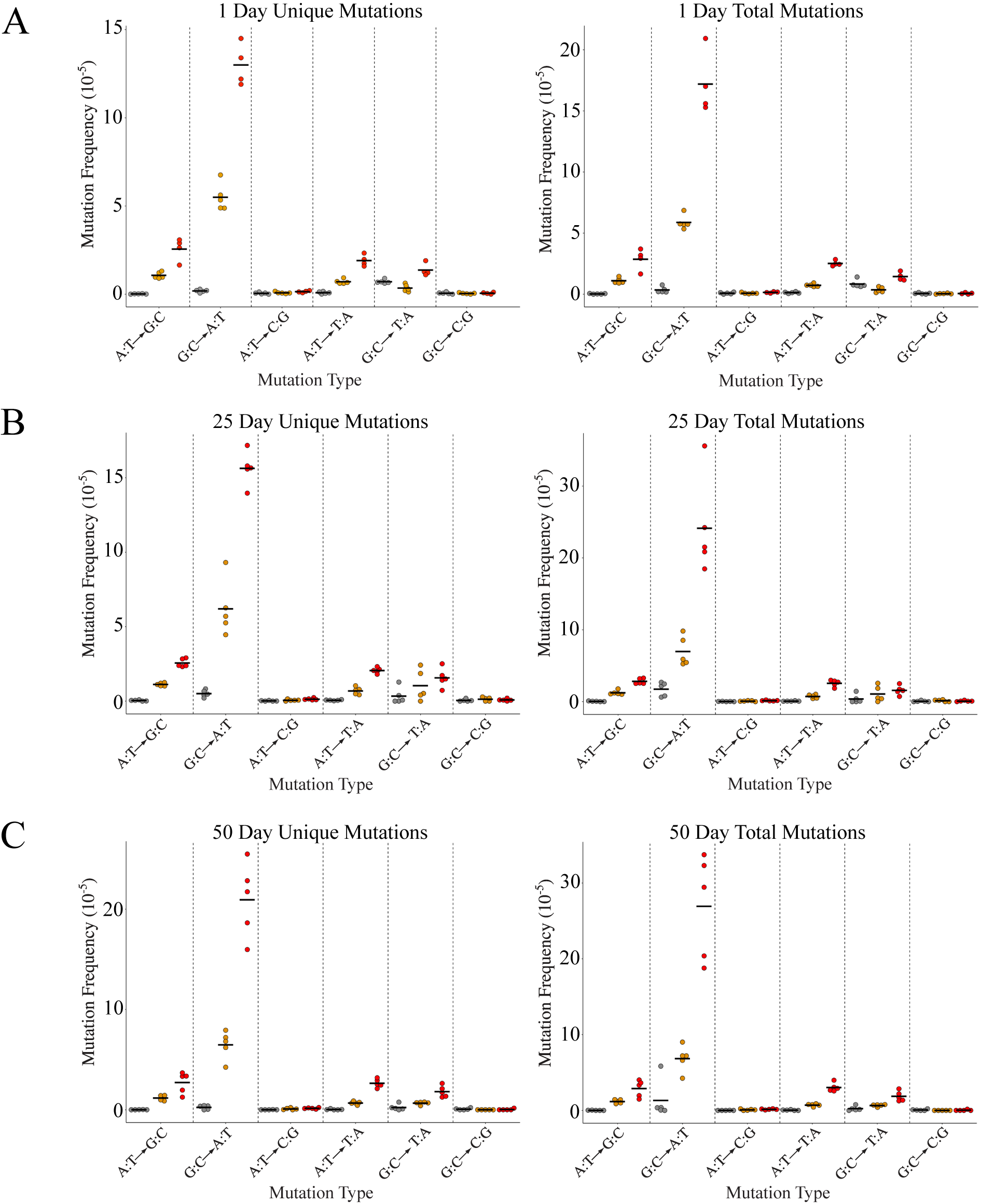

Supplement: S2 Fig — The frequency of each type of base substitution mutation for Unique and Total mutations observed in in (A) 1-day-old, (B) 25-day-old, and (C) 50-day-old flies of the indicated genotype, N = 5 flies per genotype per time point. (TIF) [file pgen.1007805.s002.tif]

0xPolG<sup>mut</sup>

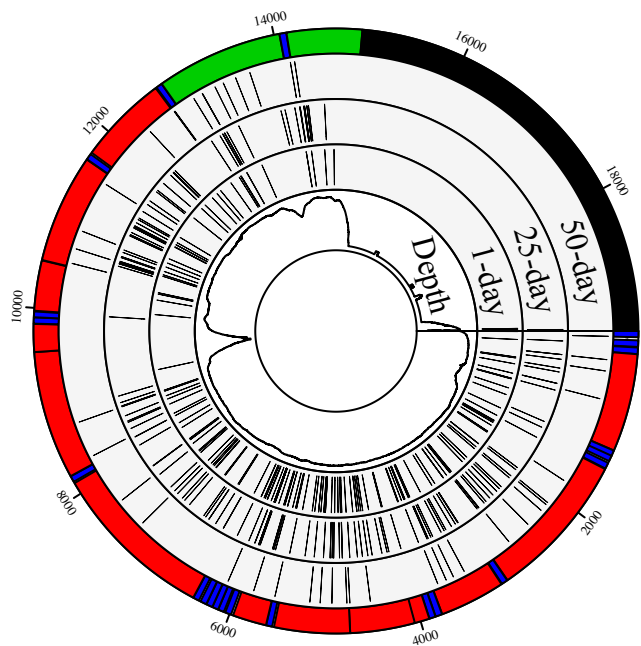

1xPolG<sup>mut</sup>

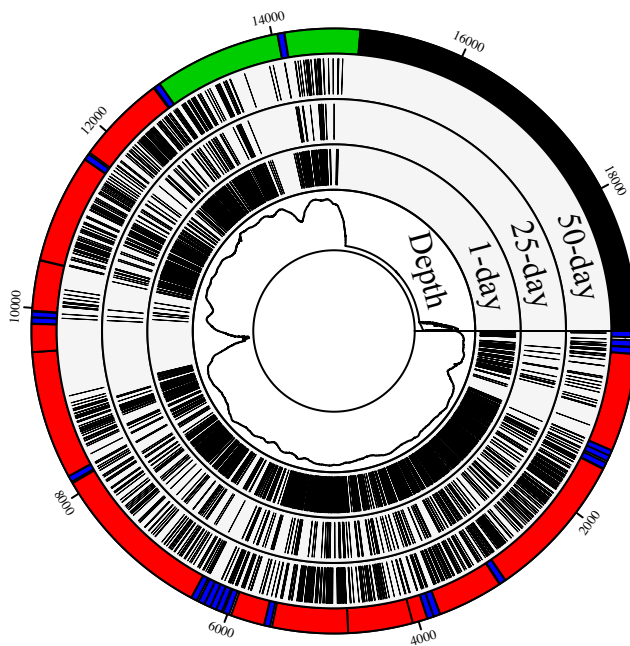

2xPolG<sup>mut</sup>

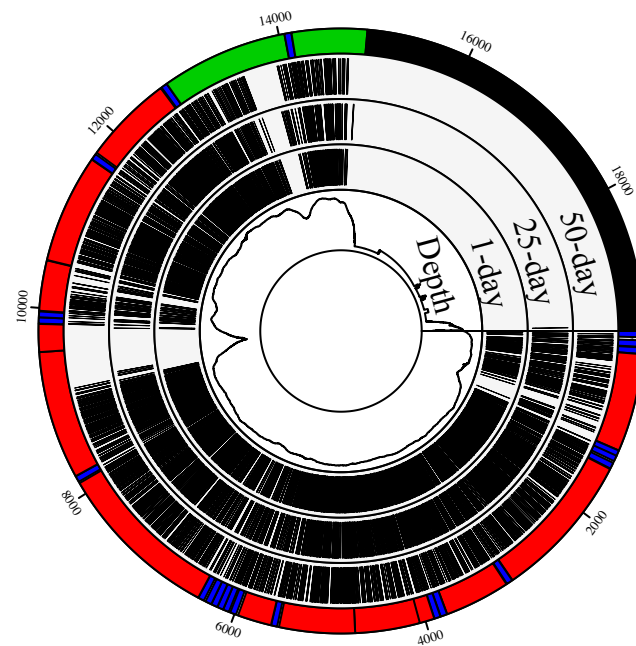

Supplement: S3 Fig — Plots of mtDNA mutations identified in 0xPolGmut, 1xPolGmut and 2xPolGmut flies. The outermost track of each plot designates functional elements within the mitochondrial genome. Red = Protein coding; Blue = tRNA; Green = rRNA; Black = Control Region. The middle track of each plot depicts the sites where mutations were observed within animals of the age indicated. The innermost track depicts the log-transformed average sequencing depth for flies of the indicated genotype. Note the absence of sequence coverage in the AT-rich control region, as well as the region between ~(ChrM ~9100–9850) not efficiently captured in our sequencing. (PDF) [file pgen.1007805.s003.pdf]

**A**

## Insertions

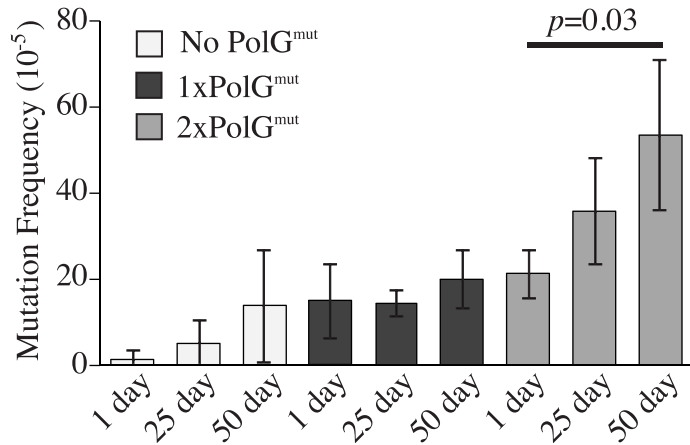**B**

## Deletions

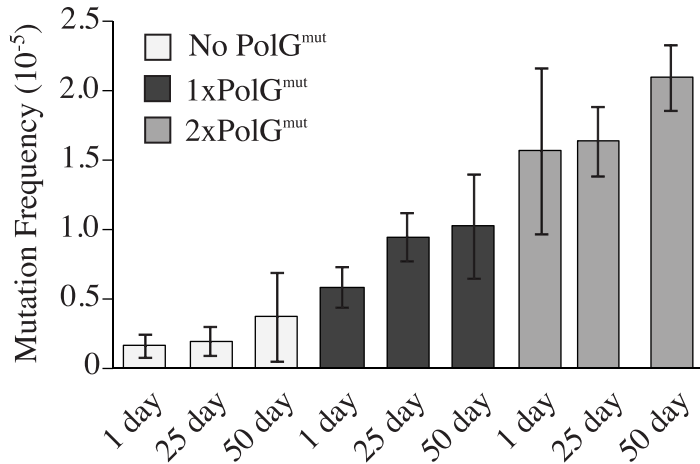

Supplement: S4 Fig — (A) The frequencies of insertion mutations were quantified in flies of the given ages and genotype using DS. (B) The frequencies of deletion mutations (≤5bp) were quantified in flies of the indicated ages and genotypes using DS. p-values determined by Student’s t-test. (PDF) [file pgen.1007805.s004.pdf]

**A** $0xPolG^{mut}$ 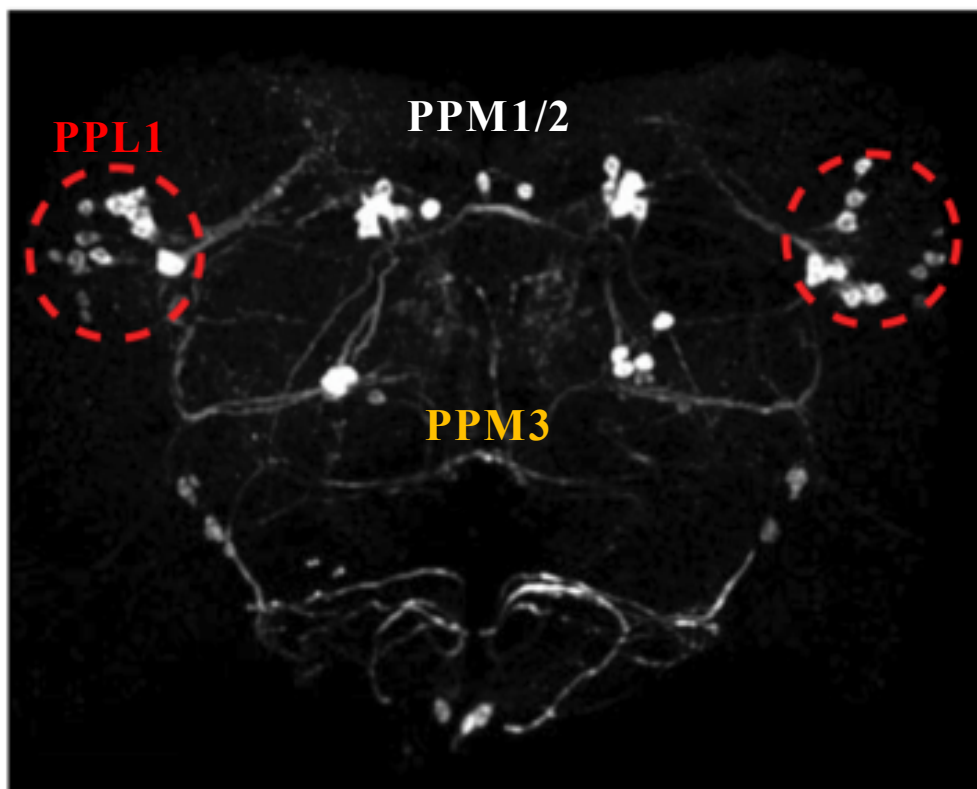**B** $2xPolG^{mut}$ 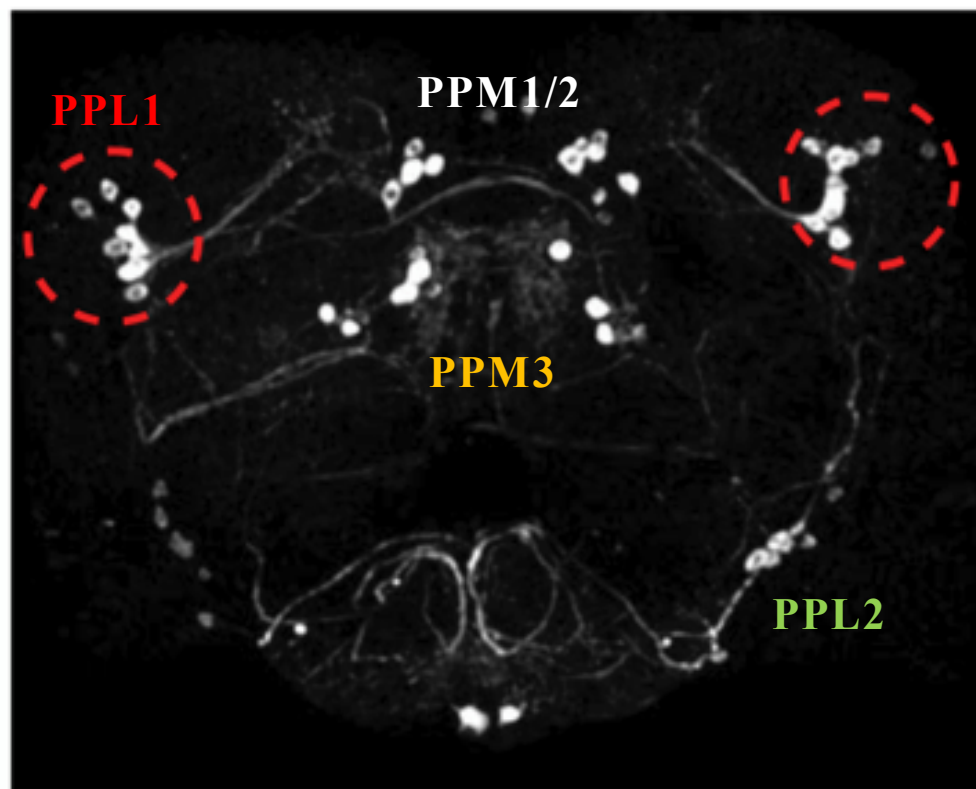

Supplement: S5 Fig — Representative confocal images of immunostained brains of 50-day-old (A) 0xPolGmut and (B) 2xPolGmut flies. Dopaminergic neurons were immunostained using tyrosine hydroxylase antiserum. The PPL1-2 and PPM1-3 clusters of dopaminergic neurons are indicated. (PDF) [file pgen.1007805.s005.pdf]

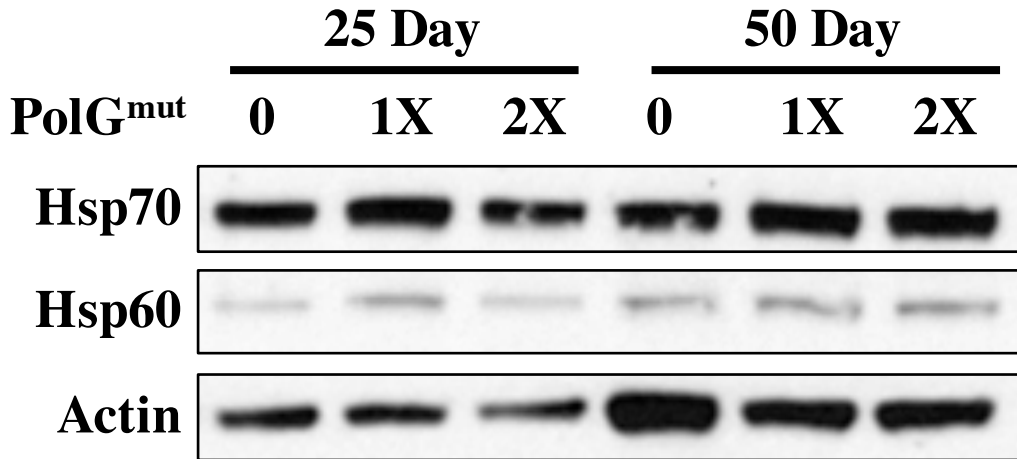

Supplement: S6 Fig — Western blots of 25- and 50-day-old 1xPolGmut (1X) and 2xPolGmut (2X) flies. Actin was used as a loading control. Images are representative of four biological replicates. (PDF) [file pgen.1007805.s006.pdf]

A

0xPolg<sup>mut</sup>

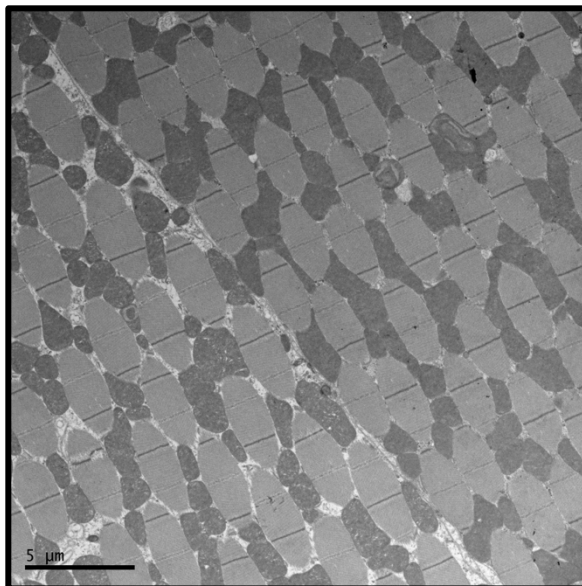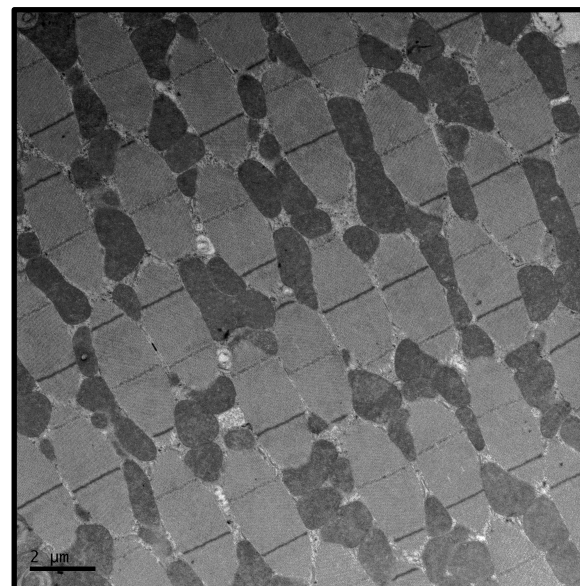

B

2xPolg<sup>mut</sup>

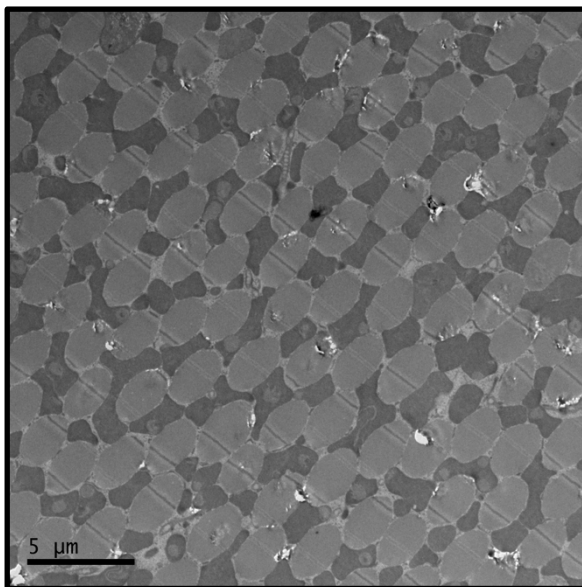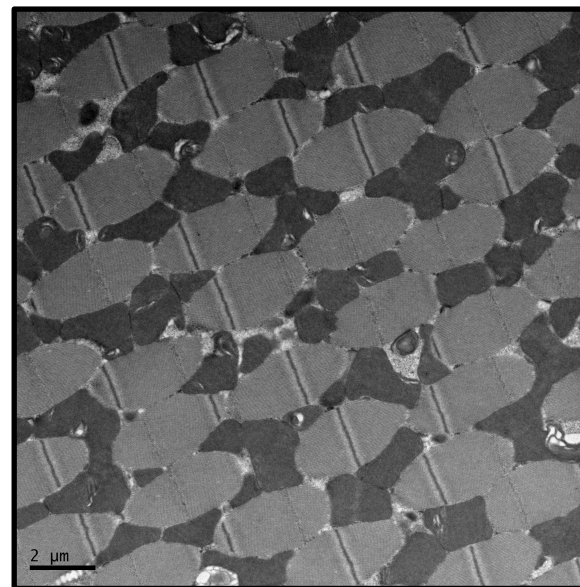

Supplement: S7 Fig — Electron microscopy of indirect flight muscle of 50-day-old (A) 0xPolgmut and (B) 2xPolgmut flies does not reveal additional mitochondrial ultrastructural defects in mutator flies. (PDF) [file pgen.1007805.s007.pdf]

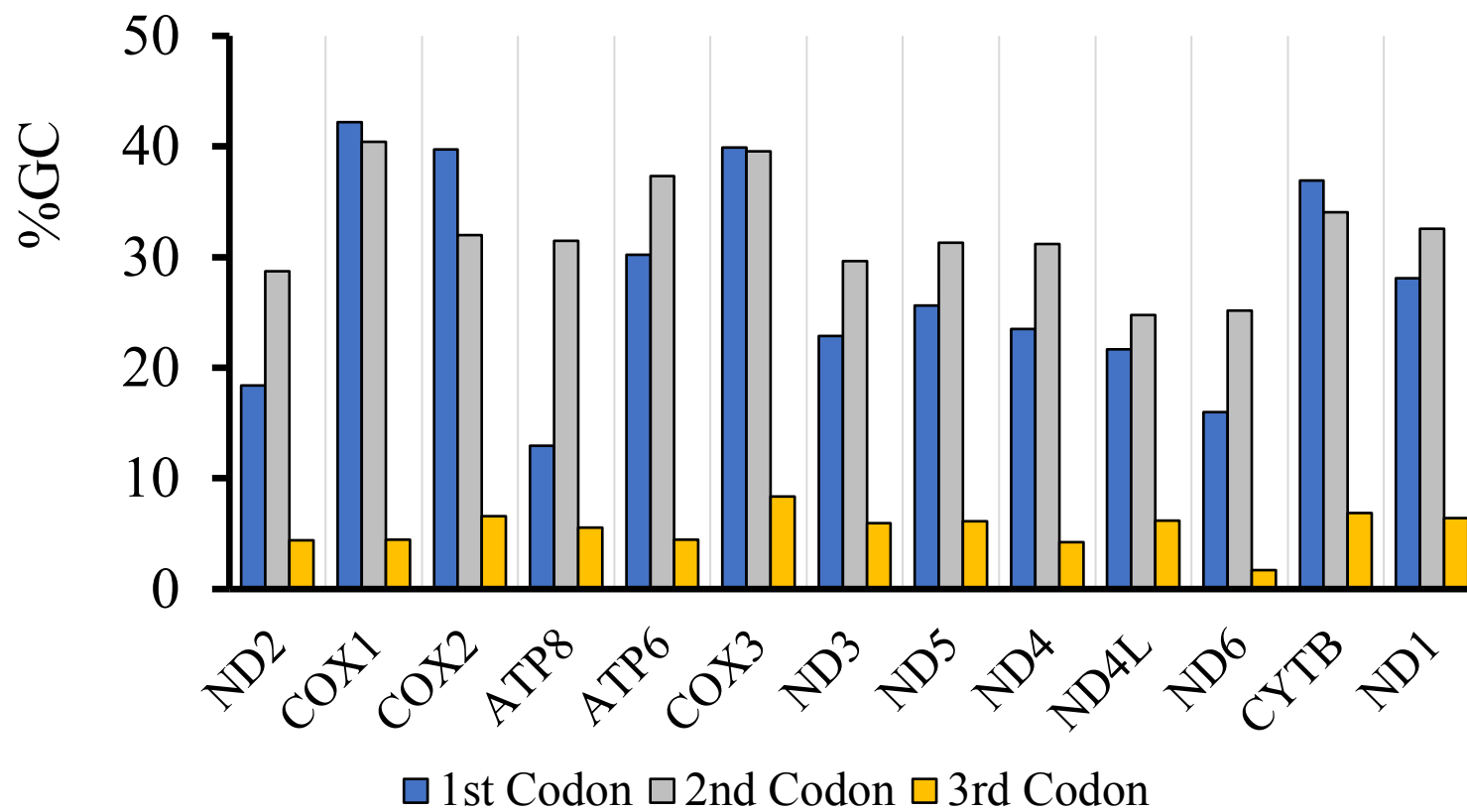

Supplement: S8 Fig — All protein-coding genes show depleted GC content in third codon positions, as calculated from the Drosophila reference genome. (PDF) [file pgen.1007805.s008.pdf]

A

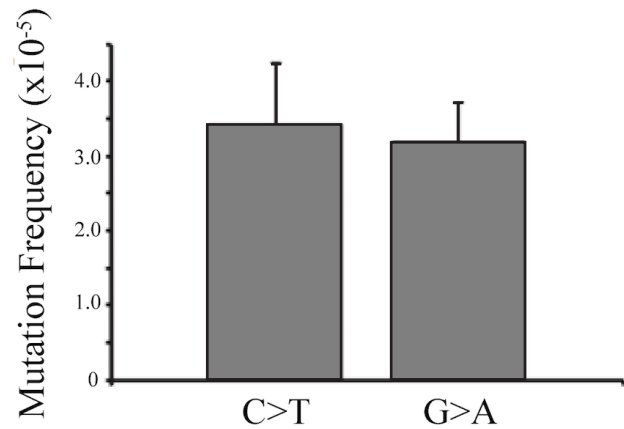

B

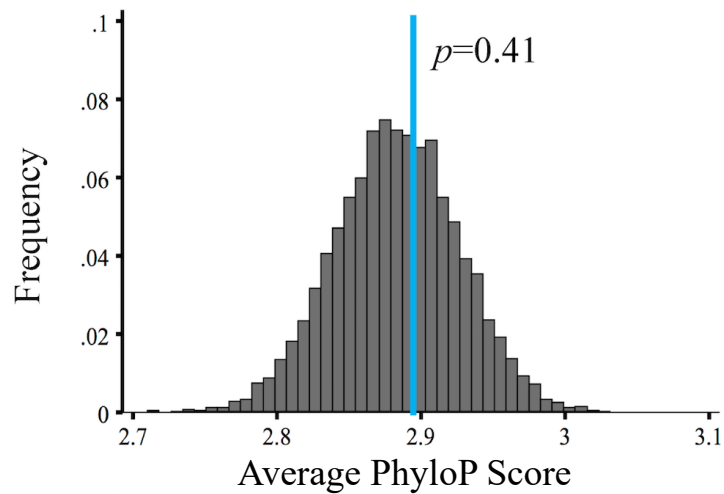

C

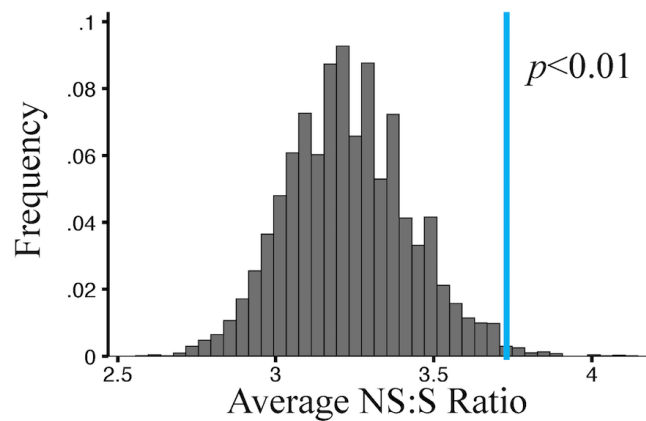

D

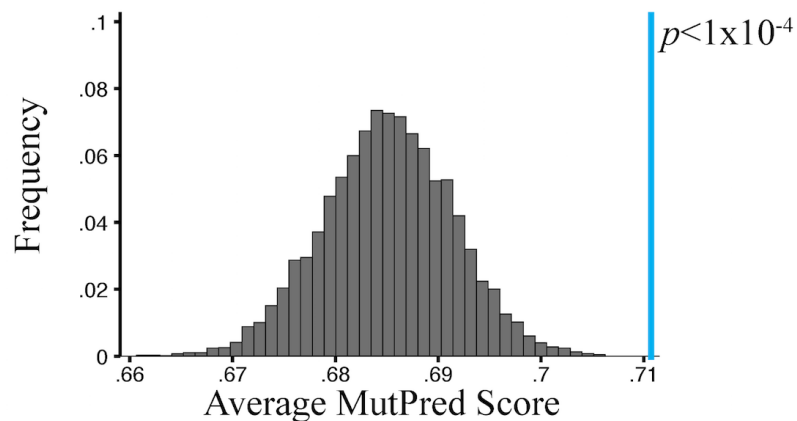

Supplement: S9 Fig — (A) Mutations within the COX1 coding sequence display no strand bias for the predominant mutation type, G:C to A:T transitions. (B) The distribution of average PhyloP scores of mutations in the COX1 coding sequence expected under neutrality, calculated from 10,000 trials of simulated mutagenesis under conditions of neutrality. Only mutations observed within the COX1 gene [ChrM 1474–3009] were used for Monte Carlo resampling in these simulations. The blue line indicates the average PhyloP score of mutations observed in the COX1 coding sequence of 1-day-old 1xPolGmut flies. (C) The distribution of average NS/S ratios of mutations in the COX1 coding sequence expected under neutrality, calculated from 10,000 simulations of random mutagenesis as described above. The blue line indicates the average NS/S ratio of mutations observed in the COX1 coding sequence of 1-day-old 1xPolGmut flies. (D) The distribution predicted average MutPred scores of NS variants observed in the Drosophila COX1 coding region under conditions of neutrality, calculated from 10,000 simulations of random mutagenesis as described above. The blue line indicates the observed average MutPred score in NS variants found in the COXI sequence in 1-day-old 1xPolGmut flies. p-values in panels B-D determined empirically. (PDF) [file pgen.1007805.s009.pdf]

**A**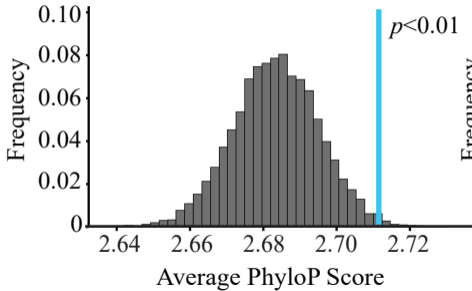**B**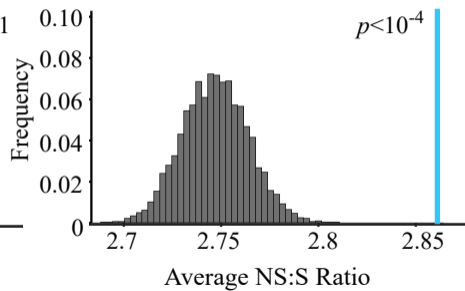**C**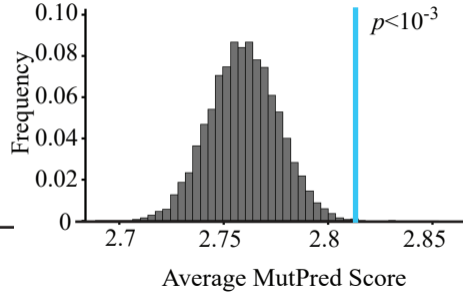

Supplement: S10 Fig — (A) The distribution of average PhyloP scores of mutations in the protein-coding sequence expected under neutrality, calculated from 10,000 trials of simulated mutagenesis under conditions of neutrality. Sequence data from 1x and 2xPolGmut flies of all ages were aggregated and Monte Carlo resampling simulations were performed as above. The blue line indicates the average PhyloP score of all mutations observed in the 1xPolGmut and 2xPolGmut flies. (B) The distribution of average NS/S ratios of mutations expected under neutrality, calculated from 10,000 simulations of random mutagenesis from the combined sequence data of 1xPolGmut and 2xPolGmut flies. The blue line indicates the average NS/S ratio of mutations observed across 1xPolGmut and 2xPolGmut flies. (C) The distribution predicted average MutPred scores of NS variants calculated from 10,000 simulations of random mutagenesis as described above. The blue line indicates the observed average MutPred score in NS variants found in 1xPolGmut and 2xPolGmut flies. p-values were determined empirically. (PDF) [file pgen.1007805.s010.pdf]
