# Supplementary material for: Deleterious mitochondrial DNA point mutations are overrepresented in Drosophila expressing a proofreading-defective DNA polymerase γ
Source: PLoS Genet. 2018 Nov 19;14(11):e1007805. doi: 10.1371/journal.pgen.1007805 (PMC6289449; doi:10.1371/journal.pgen.1007805)
Supplement: S1 Table — The total number of nucleotides sequenced, mutations identified, and genome-wide mutation frequency are shown for each fly for (A) uniquely mutated sites, and (B) total mutations. These sequencing data represent nucleotides for which sequence reads could be mapped to the Drosophila mtDNA reference sequence and duplex consensus strands could be constructed. (PDF) [file pgen.1007805.s012.pdf]

1-day-old Flies				25-day-old Flies				50-day-old Flies			
**Fly genotype**	**Nucleotides Sequenced**	**Number of Mutations**	**Mutation Frequency**	**Fly Genotype**	**Nucleotides Sequenced**	**Number of Mutations**	**Mutation Frequency**	**Fly Genotype**	**Nucleotides Sequenced**	**Number of Mutations**	**Mutation Frequency**
**0xPolG^{mut} 1**	42518691	90	2.12x10^{-6}	**0xPolG^{mut} 1**	5406269	10	1.85x10^{-6}	**0xPolG^{mut} 1**	5679645	13	2.29x10^{-6}
**0xPolG^{mut} 2**	8199467	33	4.02x10^{-6}	**0xPolG^{mut} 2**	19092032	123	6.44x10^{-6}	**0xPolG^{mut} 2**	2215855	8	3.61x10^{-6}
**0xPolG^{mut} 3**	9037683	27	2.99x10^{-6}	**0xPolG^{mut} 3**	2661304	6	2.25x10^{-6}	**0xPolG^{mut} 3**	9677840	7	7.23x10^{-7}
**0xPolG^{mut} 4**	6147590	27	4.39x10^{-6}	**0xPolG^{mut} 4**	11853341	12	1.01x10^{-6}	**0xPolG^{mut} 4**	9506293	16	1.68x10^{-6}
**0xPolG^{mut} 5**	6741158	23	3.41x10^{-6}	**0xPolG^{mut} 5**	4407413	16	3.63x10^{-6}	**0xPolG^{mut} 5**	3364513	0	<2.97x10^{-7}
**1xPolG^{mut} 1**	8070242	226	2.80x10^{-5}	**1xPolG^{mut} 1**	2450795	86	3.51x10^{-5}	**1xPolG^{mut} 1**	8110386	243	3.00x10^{-5}
**1xPolG^{mut} 2**	6288317	181	2.88x10^{-5}	**1xPolG^{mut} 2**	1542728	46	2.98x10^{-5}	**1xPolG^{mut} 2**	9385321	343	3.65x10^{-5}
**1xPolG^{mut} 3**	4066001	137	3.37x10^{-5}	**1xPolG^{mut} 3**	2015717	61	3.03x10^{-5}	**1xPolG^{mut} 3**	1788143	45	2.52x10^{-5}
**1xPolG^{mut} 4**	14242560	372	2.61x10^{-5}	**1xPolG^{mut} 4**	3447601	107	3.10x10^{-5}	**1xPolG^{mut} 4**	1204779	38	3.15x10^{-5}
**1xPolG^{mut} 5**	24762695	613	2.48x10^{-5}	**1xPolG^{mut} 5**	2938793	98	3.33x10^{-5}	**1xPolG^{mut} 5**	3081790	112	3.63x10^{-5}
**1xPolG$^{mut-Chr2}$ 1**	52868988	1186	2.24x10^{-5}	**2xPolG^{mut} 1**	5462486	404	7.40x10^{-5}	**2xPolG^{mut} 1**	2422131	242	9.99x10^{-5}
**1xPolG$^{mut-Chr2}$ 2**	65209719	1526	2.34x10^{-5}	**2xPolG^{mut} 2**	6547279	517	7.90x10^{-5}	**2xPolG^{mut} 2**	1896758	188	9.91x10^{-5}
**1xPolG$^{mut-Chr2}$ 3**	70944805	1480	2.09x10^{-5}	**2xPolG^{mut} 3**	4274349	333	7.79x10^{-5}	**2xPolG^{mut} 3**	3432156	375	1.09x10^{-4}
**1xPolG$^{mut-Chr2}$ 4**	74409046	1688	2.27x10^{-5}	**2xPolG^{mut} 4**	7056676	553	7.84x10^{-5}	**2xPolG^{mut} 4**	6967151	594	8.53x10^{-5}
**2xPolG^{mut} 1**	14409626	967	6.71x10^{-5}	**2xPolG^{mut} 5**	7133239	505	7.08x10^{-5}	**2xPolG^{mut} 5**	5222775	430	8.23x10^{-5}
**2xPolG^{mut} 2**	1951359	159	8.15x10^{-5}								
**2xPolG^{mut} 3**	14911301	951	6.38x10^{-5}								
**2xPolG^{mut} 4**	10731036	675	6.29x10^{-5}								

1-day-old Flies				25-day-old Flies				50-day-old Flies			
Fly genotype	Nucleotides Sequenced	Number of Mutations	Mutation Frequency	Fly Genotype	Nucleotides Sequenced	Number of Mutations	Mutation Frequency	Fly Genotype	Nucleotides Sequenced	Number of Mutations	Mutation Frequency
**0x$PolG^{mut}$ 1**	42518691	111	2.61x10^{-6}	**0x$PolG^{mut}$ 1**	5406269	33	6.10x10^{-6}	**0x$PolG^{mut}$ 1**	5679645	13	2.29x10^{-6}
**0x$PolG^{mut}$ 2**	8199467	35	4.27x10^{-6}	**0x$PolG^{mut}$ 2**	19092032	222	1.16x10^{-5}	**0x$PolG^{mut}$ 2**	2215855	8	3.61x10^{-6}
**0x$PolG^{mut}$ 3**	9037683	43	4.76x10^{-6}	**0x$PolG^{mut}$ 3**	2661304	7	2.63x10^{-6}	**0x$PolG^{mut}$ 3**	9677840	11	1.14x10^{-6}
**0x$PolG^{mut}$ 4**	6147590	27	4.39x10^{-6}	**0x$PolG^{mut}$ 4**	11853341	32	2.70x10^{-6}	**0x$PolG^{mut}$ 4**	9506293	147	1.55x10^{-5}
**0x$PolG^{mut}$ 5**	6741158	51	7.57x10^{-6}	**0x$PolG^{mut}$ 5**	4407413	35	7.94x10^{-6}	**0x$PolG^{mut}$ 5**	3364513	0	$<2.97x10^{-7}$
**1x$PolG^{mut}$ 1**	8070242	240	2.97x10^{-5}	**1x$PolG^{mut}$ 1**	2450795	89	3.63x10^{-5}	**1x$PolG^{mut}$ 1**	8110386	255	3.14x10^{-5}
**1x$PolG^{mut}$ 2**	6288317	192	3.05x10^{-5}	**1x$PolG^{mut}$ 2**	1542728	46	2.98x10^{-5}	**1x$PolG^{mut}$ 2**	9385321	383	4.08x10^{-5}
**1x$PolG^{mut}$ 3**	4066001	138	3.39x10^{-5}	**1x$PolG^{mut}$ 3**	2015717	62	3.08x10^{-5}	**1x$PolG^{mut}$ 3**	1788143	45	2.52x10^{-5}
**1x$PolG^{mut}$ 4**	14242560	398	2.79x10^{-5}	**1x$PolG^{mut}$ 4**	3447601	134	3.89x10^{-5}	**1x$PolG^{mut}$ 4**	1204779	38	3.15x10^{-5}
**1x$PolG^{mut}$ 5**	24762695	709	2.86x10^{-5}	**1x$PolG^{mut}$ 5**	2938793	118	4.02x10^{-5}	**1x$PolG^{mut}$ 5**	3081790	116	3.76x10^{-5}
**1x$PolG^{mut-Chr2}$ 1**	52868988	1686	3.19x10^{-5}	**2x$PolG^{mut}$ 1**	5462486	476	8.71x10^{-5}	**2x$PolG^{mut}$ 1**	2422131	267	1.10x10^{-4}
**1x$PolG^{mut-Chr2}$ 2**	65209719	3052	4.68x10^{-5}	**2x$PolG^{mut}$ 2**	6547279	872	1.33x10^{-4}	**2x$PolG^{mut}$ 2**	1896758	200	1.05x10^{-4}
**1x$PolG^{mut-Chr2}$ 3**	70944805	1742	2.46x10^{-5}	**2x$PolG^{mut}$ 3**	4274349	461	1.08x10^{-4}	**2x$PolG^{mut}$ 3**	3432156	496	1.45x10^{-4}
**1x$PolG^{mut-Chr2}$ 4**	74409046	2094	2.81x10^{-5}	**2x$PolG^{mut}$ 4**	7056676	690	9.78x10^{-5}	**2x$PolG^{mut}$ 4**	6967151	688	9.87x10^{-5}
**2x$PolG^{mut}$ 1**	14409626	1427	9.90x10^{-5}	**2x$PolG^{mut}$ 5**	7133239	654	9.17x10^{-5}	**2x$PolG^{mut}$ 5**	5222775	595	1.14x10^{-4}
**2x$PolG^{mut}$ 2**	1951359	174	8.92x10^{-5}								
**2x$PolG^{mut}$ 3**	14911301	1286	8.62x10^{-5}								
**2x$PolG^{mut}$ 4**	10731036	839	7.82x10^{-5}								
