# Supplementary material for: Deleterious mitochondrial DNA point mutations are overrepresented in Drosophila expressing a proofreading-defective DNA polymerase γ
Source: PLoS Genet. 2018 Nov 19;14(11):e1007805. doi: 10.1371/journal.pgen.1007805 (PMC6289449; doi:10.1371/journal.pgen.1007805)
Supplement: S3 Table — (PDF) [file pgen.1007805.s014.pdf]

Gene name/identifier	Biological function
CAH1 (carbonic anhydrase 1)	pH regulation
Adat1 (Adenosine deaminase-tRNA specific 1)	tRNA modifying enzyme
CG16865	Presumed splicesome component
CG16888	unknown
Sos (son of sevenless)	guanine nucleotide exchange factor
B (black)	aspartate decarboxylase
tamas (PolG)	mitochondrial DNA replication
Arpc1 (Actin-related protein 2/3 complex, subunit 1)	regulator of F-actin polymerization
Orc5 (Oigin recognition complex subunit 5)	DNA replication
GatC (Glutamyl-tRNA amidotransferase, subunit C)	mitochondrial translation
DNApol-gamma35 (DNA polymerase gamma 35kD)	mitochondrial DNA replication
RpI133 (RNA polymerase II 33kD subunit)	transcription
mRpS23 (mitochondrial ribosomal protein S23)	translation
CenG1A (Centaurin gamma 1A)	unknown
CG33307	unknown
CG33306	unknown
CG8997	unknown
CG7916	unknown
CG7953	unknown
CG7968	unknown
